# Exploring potential therapeutic targets for small cell lung cancer based on transcriptomics combined with Mendelian randomization analysis

**DOI:** 10.3389/fimmu.2024.1464259

**Published:** 2025-01-13

**Authors:** Zhicheng Liao, Pengcheng Jia, Yifan Li, Zhihui Zheng, Jizhou Zhang

**Affiliations:** Department of Medical Oncology, Wenzhou TCM Hospital of Zhejiang Chinese Medical University, Wenzhou, China

**Keywords:** small cell lung cancer, differentially expressed genes, expression quantitative trait loci, Mendelian randomization analysis, co-expressed genes, immune cell infiltration

## Abstract

**Objective:**

The main objective of this study was to explore and identify new genetic targets in small-cell lung cancer (SCLC) through transcriptomics analysis and Mendelian randomization (MR) analysis, which will help in the subsequent development of new therapeutic interventions.

**Methods:**

In this study, we extracted the SCLC dataset from the Gene Expression Omnibus (GEO) database, processed the data, and screened out differentially expressed genes (DEGs) using R software. Based on expression quantitative trait loci data and the genome-wide association study data of SCLC, MR analysis was used to screen the genes closely related to SCLC disease, which intersect with DEGs to obtain co-expressed genes (CEGs), and the biological functions and pathways of CEGs were further explored by enrichment analysis. In addition, the CIBERSORT algorithm was applied to assess the level of immune cell infiltration in SCLC and to analyze the correlation between CEGs and immune cells. Meanwhile, we performed a survival analysis on these five CEGs using an independent cohort of SCLC patients. Finally, the results for the target genes were validated.

**Results:**

In this study, 857 DEGs were identified, including 443 up-regulated and 414 down-regulated genes, and 5 CEGs (*PSAT1, PSRC1, COLEC12, PLLP, HP*) that were significantly associated with SCLC were identified through further intersecting. The results of enrichment analyses indicated that CEGs play important roles in several key functions and pathways. Immune-cell-related analysis revealed the unique distribution of immune cell infiltration in SCLC and the mechanism of immune cell regulation by CEGs. Survival analysis results indicated that *PSRC1* was significantly correlated with the overall survival of SCLC, and the survival rate of the high-expression group was markedly lower than that of the low-expression group. Finally, the consistency of the results between the validation group analyses and MR analysis confirmed that the results of this study is reliable.

**Conclusion:**

The CEGs and their associated functions and pathways screened in this study may be potential targets of therapeutic intervention in SCLC by targeting specific molecular pathways.

## 1 Introduction

Small-cell lung cancer (SCLC) is a high-grade neuroendocrine cancer with extremely high mortality and poor prognosis, accounting for about 13-15% of all lung cancer cases, with a high tendency to metastasize early, aggressive proliferation and easy to recur, and can be divided into two types: limited-stage and extensive-stage, of which 80-85% of SCLC patients are diagnosed with extensive-stage small-cell lung cancer (ES-SCLC) (1, 2). Currently chemoradiation is the key intervention in the therapeutic management of SCLC. SCLC has a good initial sensitivity to chemoradiation compared to other treatments, however, this response is mostly short-lived and patients are prone to recurrent diseases (1, 3). Whereas the emergence of immune checkpoint inhibitors in recent years has altered the clinical outcome of ES-SCLC patients to some extent, and the regimen of platinum-based chemotherapy in combination with anti-programmed cell death protein 1 and its ligand 1 has increased the survival of ES-SCLC patients and represents the current standard of first-line therapy, the overall survival of ES-SCLC patients is still very limited (4, 5). More research is urgently needed to explore effective therapeutic alternatives for SCLC. However, the complex and unclear mechanisms of SCLC development pose a serious challenge to studying therapeutic options. Therefore, an in-depth understanding of the molecular basis of SCLC, exploration of the complex mechanisms of SCLC, and identification of potential therapeutic targets are of great importance for the subsequent treatment of SCLC.

Mendelian randomization (MR) analysis is a method that uses genetic variation as instrumental variables (IVs) to analyze the causal relationship between exposure factors and disease, which can circumvent the reverse causality and environmental confounding inherent in traditional epidemiological methods (6, 7). In this study, we extracted SCLC microarray datasets from the Gene Expression Omnibus (GEO) database and screened out differentially expressed genes (DEGs) between SCLC and normal tissue samples. Based on the expression quantitative trait loci data and genome-wide association study (GWAS) data of SCLC, we used MR analysis to screen out the genes closely related to SCLC disease and intersected them with DEGs to obtain co-expressed genes (CEGs). Gene Ontology (GO)/Kyoto Encyclopedia of Genes and Genome (KEGG) enrichment analyses were further applied to explore the biological functions and pathways of CEGs. In addition, the CIBERSORT algorithm was performed to assess the level of immune cell infiltration in SCLC and to analyze the correlation between CEGs and immune cells. We further explored the activity levels of relevant functions and pathways in specific gene expression groups by Gene Set Enrichment Analysis (GSEA). Finally, validation group analysis was performed to increase the reliability of the results of this study.

## 2 Materials and methods

### 2.1 GEO data collection

The inclusion criteria for the SCLC microarray datasets were (1) the datasets contained at least 8 samples (at least 4 SCLC samples and 4 normal tissue samples) (2); the datasets did not contain any chemically treated or genetically modified samples (3); the raw data or array gene expression profiling data must be available. In this study, three SCLC microarray datasets were extracted according to the inclusion criteria (GSE43346, GSE73160 and GSE149507, respectively), which contained a total of 107 SCLC samples and 66 control samples (detailed information can be accessed from **Table 1**), and all the gene expression matrices and corresponding platform probe annotations were available from GEO database (https://www.ncbi.nlm.nih.gov/geo/).

**Table 1:** The information of the three GEO datasets.

ID	N cases	N controls	Platforms	Experiment type	Last update date
GSE43346	25	43	GPL570	Array	Mar 25, 2019
GSE73160	64	5	GPL11028	Array	Jul 07, 2020
GSE149507	18	18	GPL23270	Array	Mar 30, 2021

### 2.2 Methodology

#### 2.2.1 Identification of DEGs

The three datasets were read, preprocessed, and calibrated individually using the R software (version 4.3.3), then combined into one total dataset and batch-corrected, and normalized and standardized using gene expression matrices and annotation files from the GEO database. To eliminate the batch effect, principal component analysis was performed on the total processed dataset using the “prcomp” function, and the results were visualized. DEGs were identified using the classical Bayesian data analysis method in the “limma” package (7). The filtering conditions were set to corrected P-value < 0.05 and LogFoldChange (LogFC) > 1, and heatmaps and volcano maps of DEGs were generated using the “peatmap” package.

#### 2.2.2 Exposure factors and outcome variables

In this study, we extracted the expression quantitative trait loci data of the Võsa U team’s study from the IEU open GWAS database (https://gwas.mrcieu.ac.uk/) as an exposure factor (8). Then we screened for single nucleotide polymorphisms (SNPs) that met the following criteria (1): strong correlation with the exposure factor (P < 5e-08) (2), no linkage disequilibrium (parameters set to r2 < 0.001, kb=10000) (3), To exclude the SNPs with weak associations or insufficient explanation of phenotypic variance, the F-value of each SNP needs to be >10 [F= β^2^/SE^2^ (β stands for the allelic effect value and SE stands for standard error)]. Screened SNPs will be analyzed by MR as IVs representing exposure factors.

We extracted the GWAS data of the McKay team’s study from the GWAS catalog database (https://www.ebi.ac.uk/gwas/home, accession number GCST004746), containing a total of 2,664 SCLC samples with 21,444 control samples, which will be analyzed as an outcome variable for MR (9).

#### 2.2.3 MR analysis

In this study, MR analyses were conducted between IVs and outcome variables using the “TwoSampleMR” package in R software. Inverse variance weighting (IVW) was used as the primary analytical method, and MR Egger, weighted median, simple mode and weighted mode were used as secondary analytical methods (7). Genes that met the following criteria were screened based on the MR analysis results (1): IVW results showed P-value <0.05 (2); the results of the above five analysis methods met the criterion of directional consistency [Odds ratios (ORs) were directionally consistent] (3); the IVW results were corrected using the false discovery rate method (the adjusted P-value was <0.05) (4); the pleiotropic analysis results showed no pleiotropic tendency (P>0.05). According to the direction of OR value, the screened genes were respectively intersected and identified with DEGs thereby obtaining CEGs, including up-regulated and down-regulated genes. In addition, heterogeneity tests, pleiotropic and sensitivity analyses were performed to determine the reliability of the MR results. Funnel, scatter and leave-one-out plots were developed to visualize and support the MR results.

#### 2.2.4 GO/KEGG enrichment analysis

To further explore the potential biological functions and pathways of CEGs, we performed GO functional annotation and KEGG pathway enrichment analyses of the CEGs using the “clusterProfiler” package with a filter of P<0.05.

#### 2.2.5 Analysis of immune cell infiltration

In this study, the “CIBERSORT” package was performed to analyze and assess the differences in the infiltration of 22 immune cells between SCLC and normal tissue samples, and to further analyze the correlation between CEGs and immune cells for exploring the mechanism of CEGs on immune cells (10).

#### 2.2.6 GSEA

GSEA can sort all genes from largest to smallest according to the multiplicity of differences after treatment, and if there is a trend of up-regulation or down-regulation of functions and pathways associated with gene expression then it will show top or bottom enrichment in the gene list (11). Therefore, in this study, we further used the GSEA method to explore the activity levels of the relevant functions and pathways in a specific gene set with a statistical significance criterion of P<0.05.

#### 2.2.7 Survival analysis

This study retrieved the overall survival data of SCLC from the George team (12). After excluding the missing values, the data was calibrated and processed. Subsequently, the “survival” package was utilized to perform survival analysis on CEGs, thereby obtaining genes associated with SCLC overall survival (with a significance criterion of P < 0.05).

#### 2.2.8 Validation group analysis

In this study, the GSE40275 dataset, which met the inclusion criteria, was selected from the GEO database as the validation group (15 SCLC and 43 normal tissue samples), and was processed and analyzed using the same methodology as described above, as well as the results of the analyses were compared with the results of the MR analyses, to verify whether there were differences in the CEGs between the SCLC and normal tissue samples.

## 3 Results

### 3.1 Identification of DEGs

**Figure 1:**
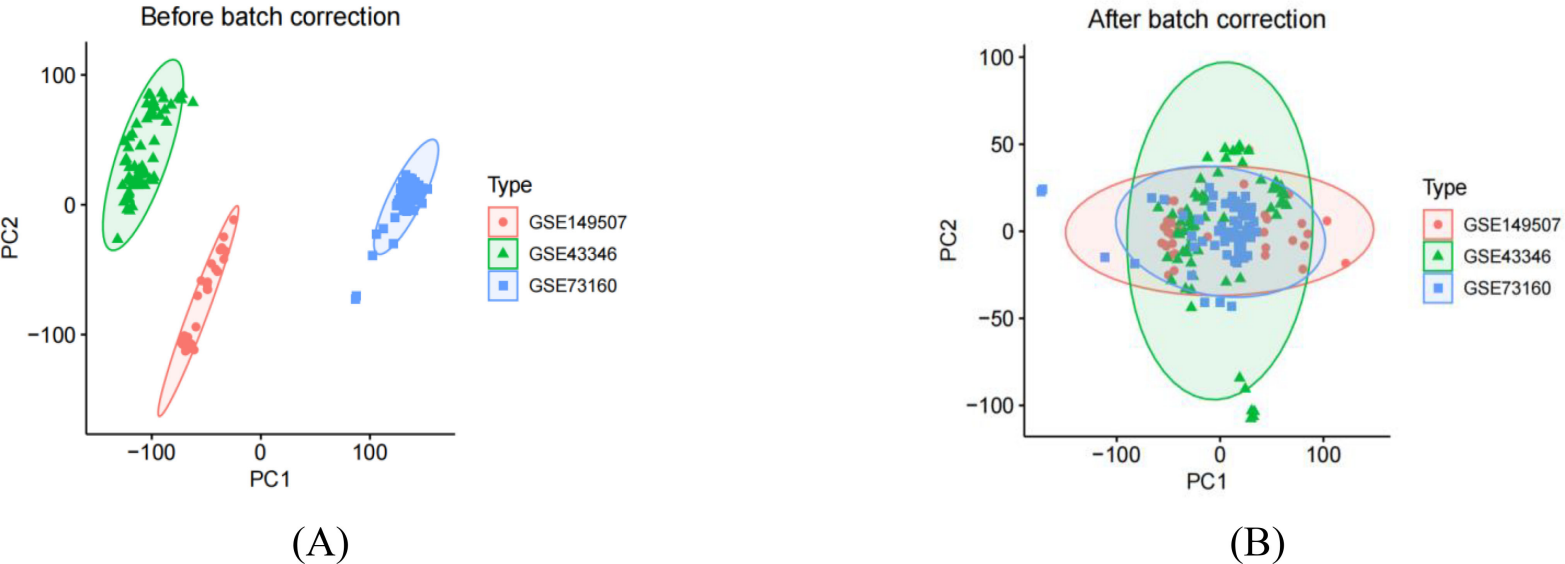
Principal component analysis of principal component analysis of three small cell lung cancer datasets. **(A)** represents that there are clear batch effects in the three datasets before batch correction. **(B)** represents, after batch correction, all samples in the dataset achieved acceptable homogeneity following PCA analysis.

**Figure 2:**
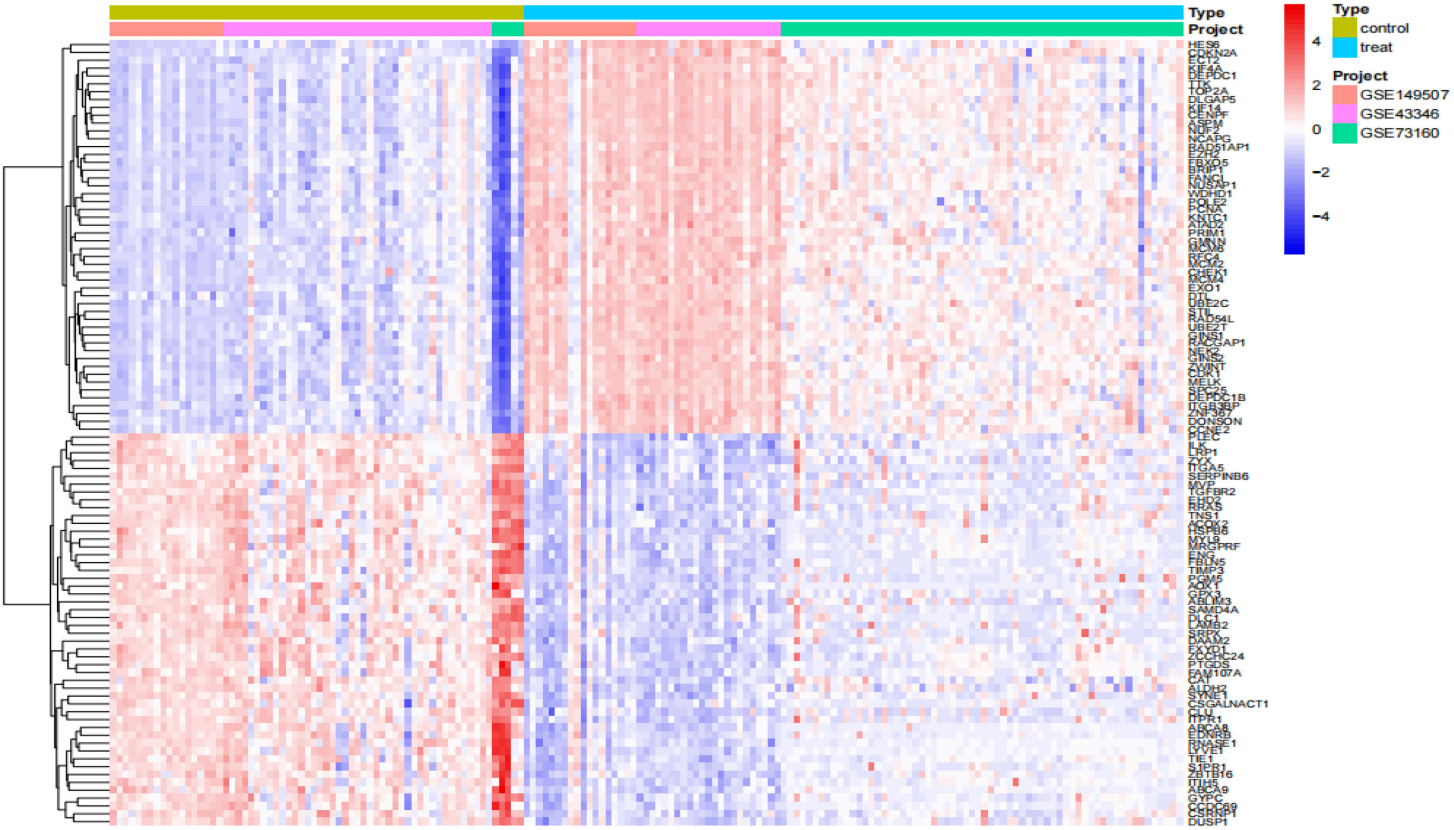
Differential gene expression heatmap. Treat: small-cell lung cancer tissue samples, control: normal tissue samples.

**Figure 3:**
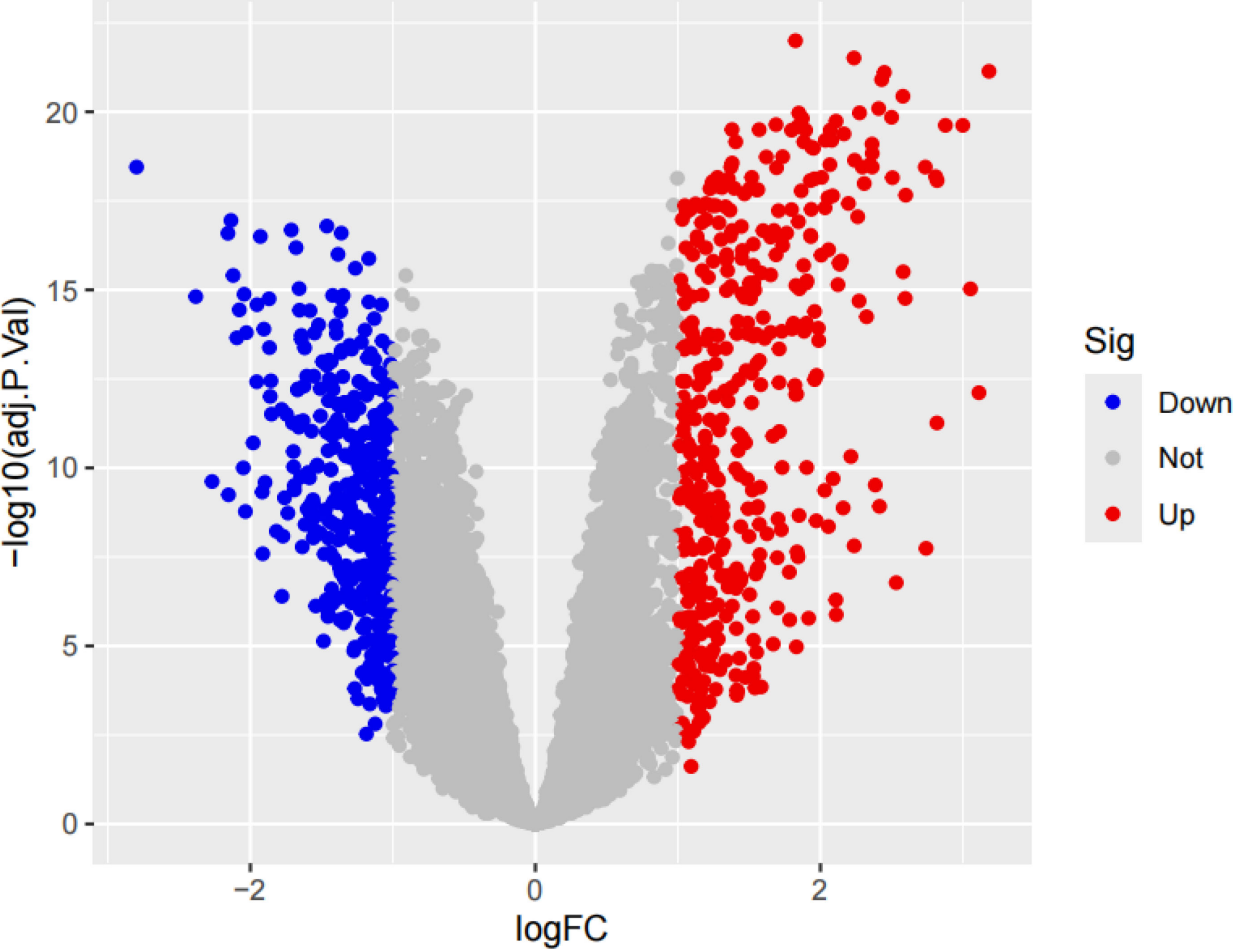
The volcano plot of DEGs with the three datasets. If the log fold change (logFC) is greater than 1, it represents an up-regulated differentially expressed gene, which is the red part; if the logFC is less than -1, it represents a down-regulated differentially expressed gene, which is the blue part.

We performed a series of pre-processing on each of the three datasets and corrected the expression of each gene in the data, and finally merged them into one total dataset and removed the batch effect by principal component analysis. The data before and after batch correction are shown in **Figures 1A, B**. In this study, 857 DEGs were identified from the three GEO datasets, including 443 up-regulated genes and 414 down-regulated genes (specific information can be obtained from **Supplementary Table S1**). The heatmap shows the expression status of the top 50 up-regulated DEGs and the top 50 down-regulated DEGs, as shown in **Figure 2**. The volcano plot shows the expression pattern of DEGs in the total dataset, as shown in **Figure 3**.

### 3.2 MR analysis

**Figure 4:**
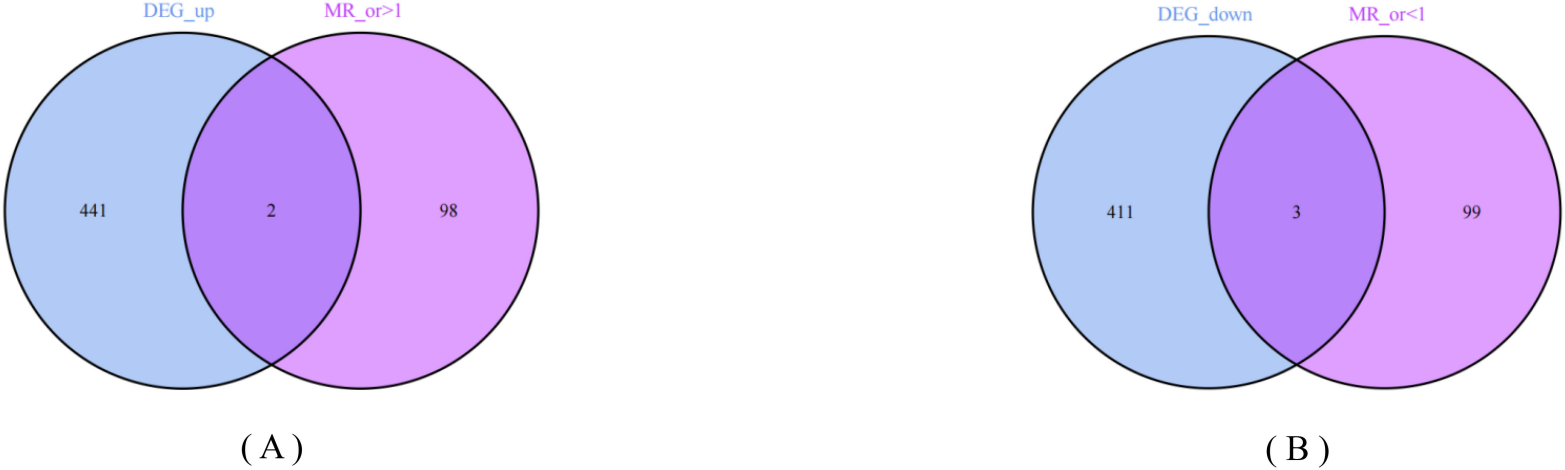
Venn plots. Two circles respectively represent the differential expression analysis results and Mendelian randomization analysis results of small cell lung cancer. The overlapping part represents the co-expressed genes. **(A)** 2 up-regulated co-expressed genes. **(B)** 3 down-regulated co-expressed genes.

Based on the three main hypothesis criteria of MR analysis and the F-value filtering criteria (7, 13), we finally filtered 26,512 SNPs as instrumental variables (specific information can be obtained from **Supplementary Table S2**). MR analysis was performed between IVs and outcome variables. According to the filtering criteria, we filtered out 102 genes with OR-value <1 and 100 genes with OR-value >1 (specific information can be obtained from **Supplementary Table S3**), further intersected with DEGs, and finally obtained 2 co-expression up-regulated genes (*PSAT1*, *PSRC1*) and 3 co-expression down-regulated genes (*PLLP*, *COLEC12*, *HP*), as shown in **Figure 4**.

**Figure 5:**
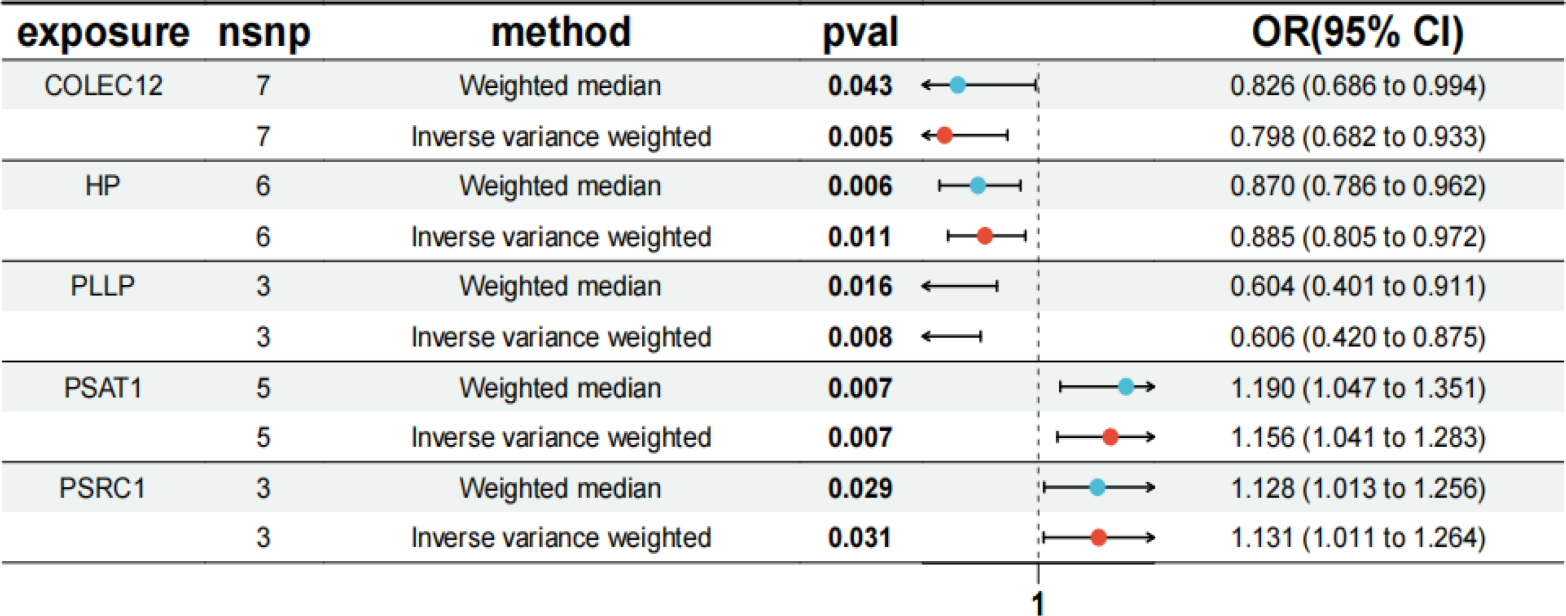
MR forest plot of co-expressed genes. The results of MR analysis between co-expressed genes and SCLC disease. OR, odds ratio; CI, confidence interval; snp, single-nucleotide polymorphism.

**Figure 6:**
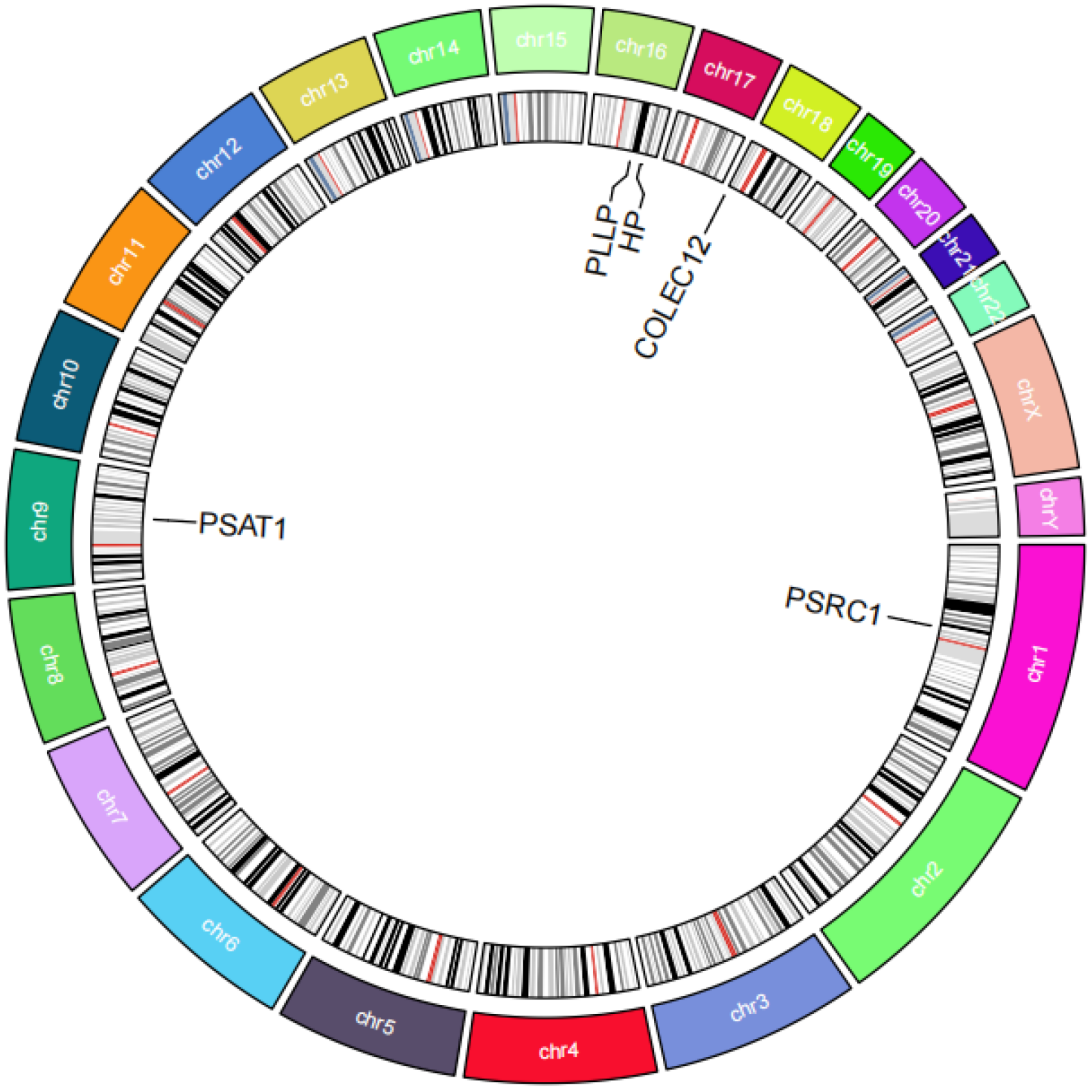
Circos plot of co-expressed genes. The figure shows a circular chromosome diagram. The regions of different colors in the diagram represent different chromosomes, and the locations of five co-expressed genes on the chromosomes are labeled.

The IVW results showed that *PSAT1* and *PSRC1* all had significant positive correlation with SCLC, *PSAT1* [P=0.007, OR=1.156, 95% confidence interval (CI): 1.041-1.283], *PSRC1* (P=0.031, OR=1.131, 95% CI: 1.011-1.264); whereas *COLEC12*, *PLLP*, and *HP* all had significant negative correlation with SCLC, *COLEC12* (P=0.005, OR=0.798, 95% CI: 0.682-0.933), *PLLP* (P=0.008, OR=0.606, 95% CI: 0.420-0.875), *HP* (P=0.011, OR=0.885, 95% CI: 0.805-0.972), as shown in **Figure 5**. In the results of the analysis of multiplicity all P-values were >0.05, indicating that there was no effect of multiplicity. In the test for heterogeneity all p-values were >0.05, indicating that there was no effect of heterogeneity. Results of leave-one-out sensitivity analyses showed that the effect value of each IV were close to the overall effect size. Forest, scatter, funnel, and leave-one-out plots can be obtained from **Supplementary Figure S1**. In addition, we localized the CEGs on chromosomes and visualized them as shown in **Figure 6**.

### 3.3 GO/KEGG enrichment analysis

**Figure 7:**
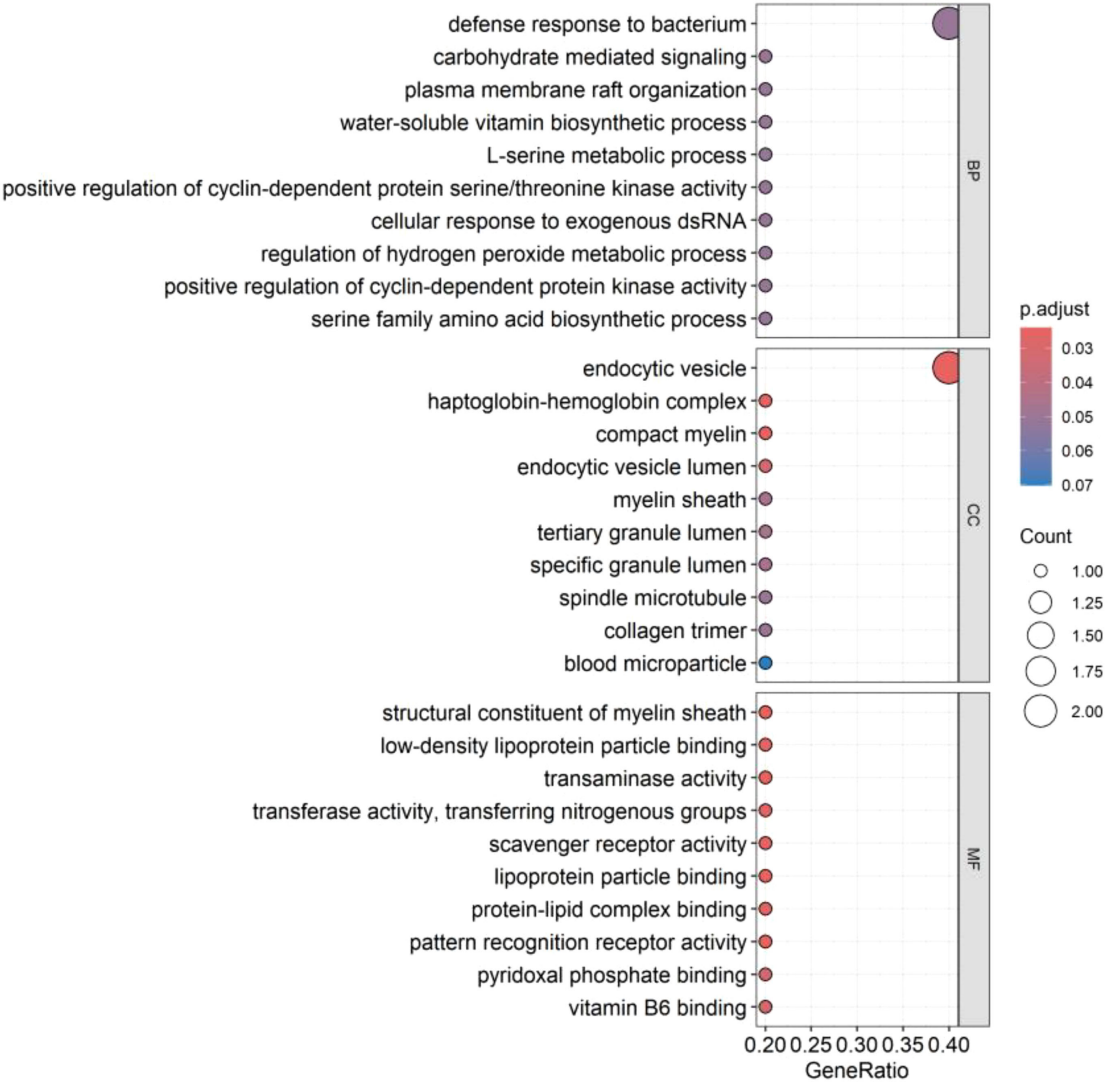
GO enrichment analysis of co-expressed genes. The figure illustrates the functional enrichment status of co-expressed genes within the GO enrichment analysis, covering biological process (BP), cellular component (CC), and molecular function (MF). Each segment showcases the top ten functions. A deeper red color in “p.adjust” indicates a higher level of significance. The white circles signify the quantity of genes where genes are concentrated within a particular function. Larger circles denote a greater number of enriched genes.

**Figure 8:**
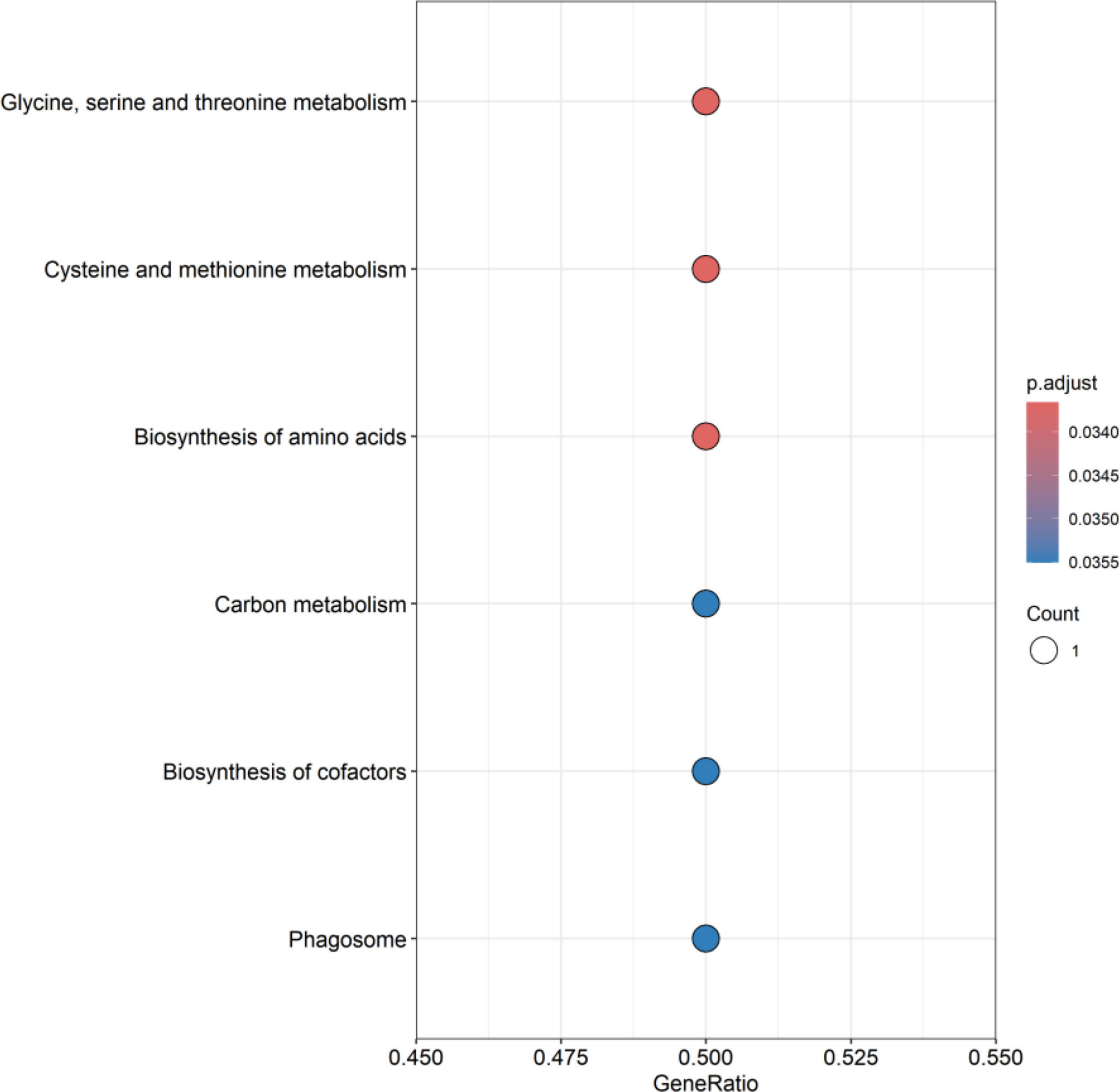
KEGG enrichment analysis of co-expressed genes. This figure presents the pathway enrichment status of co-expressed genes in KEGG enrichment analysis, showing the top ten pathways. A deeper red color in “p.adjust” indicates a higher level of significance. The white circles represent the number of genes where differentially expressed genes (DEG) are concentrated in a specific pathway. Larger circles imply a greater number of enriched genes.

GO enrichment analysis showed that these genes mainly affect the biological functions of endocytic vesicle, haptoglobin-hemoglobin complex, compact myelin, myelin sheath, structural constituent of myelin sheath, as shown in **Figure 7**. KEGG enrichment analysis showed that these genes mainly affect glycine, serine, and threonine metabolism; cysteine and methionine metabolism; biosynthesis of amino acids; carbon metabolism, biosynthesis of cofactors, and phagosome, as shown in **Figure 8**. Specific information can be obtained from the **Supplementary Table S7**.

### 3.4 Immune cell infiltration analysis

**Figure 9:**
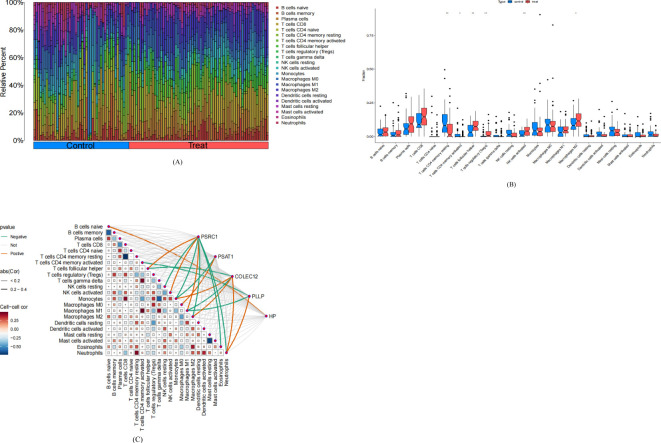
Analysis of Immune Cell Infiltration in small-cell lung cancer. Treat: small-cell lung cancer group, control: normal tissue group. **(A)** Stacked histogram of the ratio of immune cells in the treat and control groups. **(B)** Box plots comparing the levels of 22 types of immune cell infiltration in the treat group versus the control group. *P<0.05, **P<0.01. **(C)** Heatmap of the correlation between co-expressed genes and 22 types of immune cell.

The results of GO/KEGG enrichment analysis showed that the functions and pathways of CEGs may be related to immune infiltration; therefore, in this study, we used the CIBERSORT algorithm to assess the differences in the levels of immune cell infiltration between SCLC and control samples and further explored the correlation between CEGs and immune cell infiltration. We observed significant differences between SCLC and control samples on T cells CD4 memory resting, T cells CD4 memory activated, T cells follicular helper, T cells regulatory, NK cells activated, and Macrophage M2, as shown in **Figure 9A**. Specifically, the proportion of T cell CD4 memory resting and T cell CD4 memory activated were significantly lower. T cell follicular helper, T cells regulatory, NK cell activated, and Macrophages M2 were significantly higher in SCLC compared with control, as shown in **Figure 9B**.

Correlation analysis of CEGs with 22 immune cells showed that *PSRC1* was positively correlated with T cells follicular helper, NK cells activated, Macrophages M1, and negatively correlated with NK cells resting, Monocytes, Macrophages M2, Eosinophils, and Neutrophils; *PSAT1* was positively correlated with Macrophages M0, and negatively correlated with Monocytes; *COLEC12* was positively correlated with Monocytes, Macrophages M2 and Neutrophils, and negatively correlated with T cells follicular helper and Macrophages M1; *PLLP* was positively correlated with Neutrophils and negatively correlated with T cell CD4 memory activated and Macrophages M1; *HP* was negatively correlated with B cells native, as shown in **Figure 9C**.

### 3.5 GSEA

Based on the results of differential gene expression status, immune cell infiltration assessment, and correlation analysis, we found that *PSRC1* and *COLEC12* were associated with significantly differential infiltrating immune cells. Therefore, this study further explored the activity levels of the relevant functions and pathways of *PSRC1* and *COLEC12* in SCLC using GSEA. GSEA results showed that, in the *PSRC1* high expression group, the top five active biological functions were chromosome segregation, mitotic sister chromatid segregation, nuclear chromosome segregation, regulation of chromosome segregation, and sister chromatid segregation; the top five active pathways were cell cycle, DNA replication, mismatch repair, oocyte meiosis, and P53 signaling pathway. In the *PSRC1* low-expression group, the top five active biological functions were adaptive immune response based on somatic recombination of immune receptors built from immunoglobulin superfamily domains, phagocytosis, T cell-mediated immunity, specific granule, and immune receptor activity; the top five active pathways were the complement and coagulation cascades, cytokine-cytokine receptor interaction, and drug-metabolism cytochrome P450, hematopoietic cell lineage, and metabolism of xenobiotic by cytochrome P450.

In the *COLEC12* high expression group, the top five active biological functions were humoral immune response, phagocytosis, positive regulation of Extracellular-regulated kinase 1 and Extracellular-regulated kinase 2 cascade, response to chemokine, and immune receptor activity; the top five active pathways were chemokine signaling pathway, complement and coagulation cascades, cytokine-cytokine receptor interaction, hematopoietic cell lineage, and vascular smooth muscle contraction; In the *COLEC12* low-expression, the top five active biological functions group were cell cycle checkpoint signaling, mitotic sister chromatid separation, chromosome region, chromosome centromeric region, and condensed chromosome; the top five active pathways are aminoacyl tRNA biosynthesis, cell cycle, DNA replication, protein export, and spliceosome. Details can be obtained from **Supplementary Figure S2**.

### 3.6 Survival analysis

**Figure 10:**
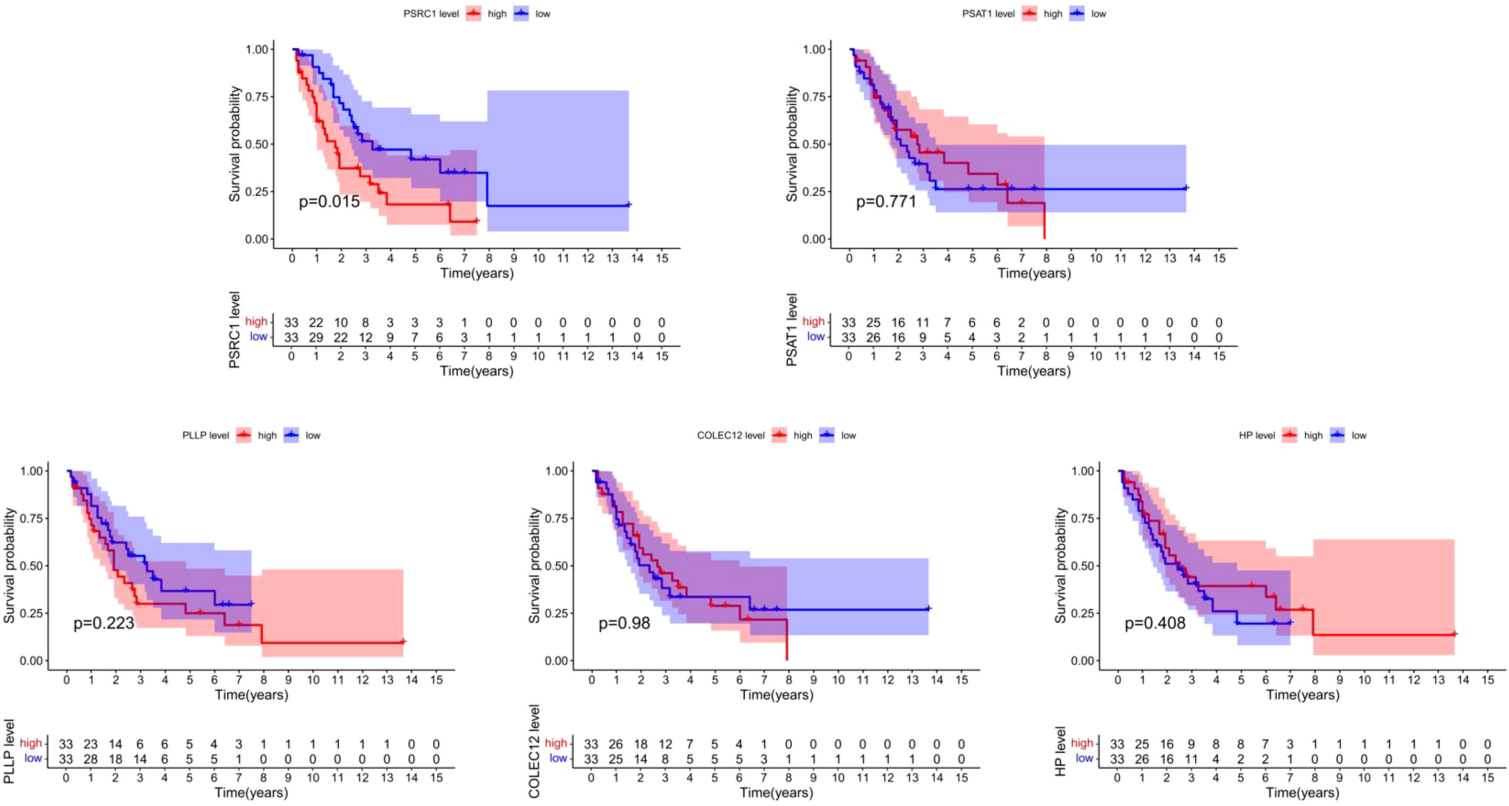
Survival analysis of co-expressed genes. The figure shows the relationship between five CEGs (*PSRC1*, *PSAT1*, *PLLP*, *COLEC12*, *HP*) and the overall survival of patients with SCLC. The red represents the group with high gene expression, and the blue represents the group with low gene expression. The horizontal axis represents overall survival time, and the vertical axis represents survival probability. The table beneath it enumerates the number of patients who remain alive at different time points spanning from 0 to 15 years.

This study extracted and sorted out the survival data of 66 patients with SCLC. Specific information can be obtained from the **Supplementary Table S8**. The results demonstrated that *PSRC1* was significantly associated with the overall survival time of SCLC patients (P = 0.015). The survival rate in the group with high expression of *PSRC1* was significantly lower than that in the group with low expression of *PSRC1*, as shown in **Figure 10**.

### 3.7 Validation group analysis

**Figure 11:**
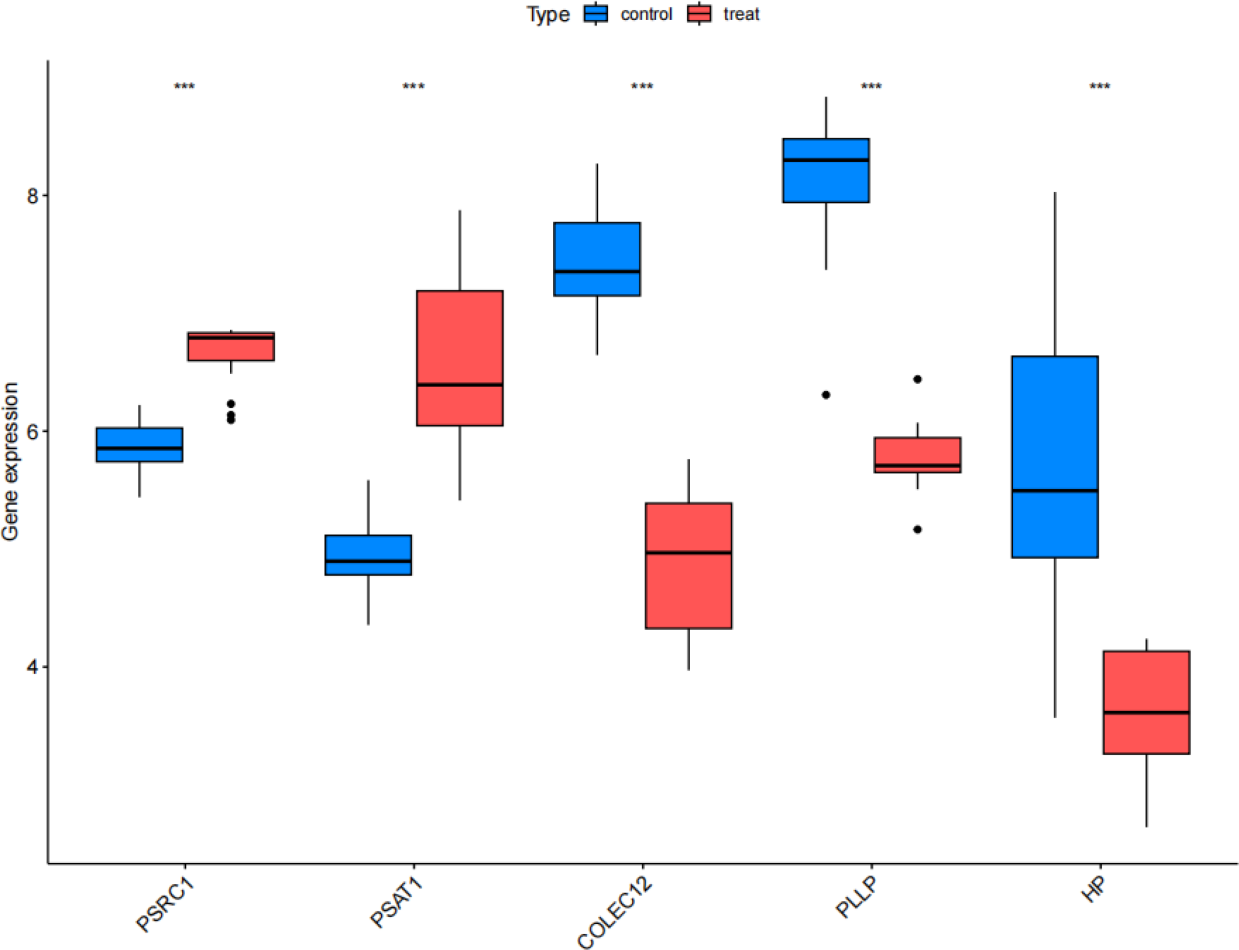
Validation group differential analysis. Box plots comparing the expression status of co-expressed genes between the treat group and control group in the validation set. Treat: small cell lung cancer group, control: normal tissue group. ***P<0.001.

The results of the validation group analysis showed a significant increase (P<0.001) in the expression of *PSAT1* and *PSRC1*, and showed a significant decrease (P<0.001) in the expression of *COLEC12*, *PLLP*, and *HP* in SCLC group, as shown in **Figure 11**. The expression levels of CEGs in the validation group were consistent with the results of this study, which increased the reliability and robust of the results of MR analysis.

## 4 Discussion

In the early stage of our research, we attempted to search for the information of each patient through the source literature of the datasets, so as to conduct a stratified analysis of factors such as age, gender, and disease stage. However, only a small portion of the datasets had information in this regard. Considering this limitation, we were worried that the research results could not fully cover every patient in the datasets. Therefore, we have not taken this aspect as the main objective of our research for the time being.

In our study, three SCLC microarray datasets were extracted from the GEO database, including 107 SCLC samples and 66 normal tissue samples. Through in-depth analysis of the datasets, we successfully identified 857 DEGs (442 up-regulated genes and 407 down-regulatedgenes) that may be critically involved in the pathophysiology of SCLC. DEGs further intersect with the closely related genes of SCLC from the results of MR analysis, we obtained two up-regulated CEGs (*PSAT1*, *PSRC1*) and three down-regulated CEGs (*COLEC12*, *PLLP*, *HP*), and MR analysis confirmed that increased expression of *PSAT1* and *PSRC1* were associated with increased risk of SCLC, while decreased expression of *COLEC12*, *PLLP*, and *HP* were associated with increased risk of SCLC, suggesting that they may play a key role in the pathogenesis of SCLC. Furthermore, an independent cohort of patients with SCLC was employed to investigate the correlation between CEGs and overall survival duration. The findings indicated that *PSRC1* was significantly related to the overall survival of SCLC patients and the survival rate in the high-expression group was markedly lower compared to that in the low-expression group, with a P-value of 0.015, suggesting a potential prognostic role of *PSRC1* expression levels in SCLC survival.

### 4.1 Up-regulated genes

*PSAT1* (Phosphoserine aminotransferase 1), located on chromosomal locus 9q21.2, is involved in the serine synthesis pathway and is a member of the class V pyridoxal phosphate-dependent aminotransferase family (14). The critical role of *PSAT1* as a key enzyme in the synthesis of serine and glycine has been verified and the overactivation of the serine/glycine metabolic pathway may promote tumorigenesis by facilitating the cell cycle process (15). Previous studies have confirmed that *PSAT1* is an oncogene that is usually over-expressed in malignant tumors, especially in non-small cell lung cancer (NSCLC) (16). Many studies have suggested that *PSAT1* may be play a key role in NSCLC, Yang et al. concluded that over-expressed *PSAT1* promoted cell cycle protein D1 activity and inhibited its degradation, thereby promoting the proliferation of NSCLC cells; Chan et al. found that over-expressed *PSAT1* promoted lung adenocarcinoma (LUAD) metastasis and led to poor prognosis by inhibiting several biological factors; Luo et al. suggested that over-expressed *PSAT1* promoted LUAD metastasis and was also responsible for resistance to epidermal growth factor receptor inhibitors in LUAD patients (15, 17, 18). The results of this study showed that *PSAT1* expression was significantly up-regulated in SCLC consistent with most malignant tumors and the results of enrichment analysis also indicated that *PSAT1* is involved in multiple pathways in the cell cycle process, such as glycine, serine and threonine metabolism. However, there are limited studies related to *PSAT1* in SCLC, our results may reveal a new research direction for an in-depth exploration of the pathogenesis of SCLC.

*PSRC1* (Proline and serine rich coiled-coil 1), located at chromosomal locus 1p13.3, is a novel microtubule-associated protein encoding proline-and serine-rich proteins and is a down-regulated target of p53 and is responsible for p53-mediated gene repression with oncogenic features (19). It has been confirmed that *PSRC1* promotes cell growth through multiple pathways, such as enhancing β-catenin dependent transactivation and cyclin D1 production by binding to adenomatous polyposis coli 2 (20). Previous studies have confirmed that *PSRC1* plays an important role in a variety of cancers, Liu et al. suggested that over-expression of *PSRC1* in patients with low-grade gliomas was an independent risk factor for shortening their overall survival; Long et al. concluded that *PSRC1* is a novel biomarker for the diagnosis and treatment of pancreatic cancer; Han et al. observed that *PSRC1* was highly expressed in LUAD and Lung squamous cell carcinoma and they concluded that high expression of *PSRC1* was an independent risk factor for overall survival and progression-free survival in patients with LUAD (21–23). The outcomes of this research have verified that *PSRC1* exhibits high expression levels in SCLC. Based on these findings, we speculate that its elevated expression may promote cell cycle progression, thereby accelerating the development of small cell lung cancer. Moreover, the survival analysis has demonstrated that the elevated expression of *PSRC1* correlates with a lower survival rate in SCLC, which aligns with the circumstances observed in non-small cell lung cancer. In view of these findings, we believe that *PSRC1* may be a potential therapeutic target for SCLC.

Interestingly, a potential link between *PSAT1* and *PSRC1* has been found in previous studies of non-small cell lung cancer, in which increased *PSAT1* expression inhibits the degradation of the cell cycle protein d1, while elevated *PSRC1* expression promotes the production of this protein (17, 20). Because of the limited research on these two genes in SCLC currently, it is still uncertain whether such a combined action exists in SCLC, yet our research may offer a new research direction.

### 4.2 Down-regulated genes

*COLEC12* (Collectin subfamily member 12), located at chromosomal locus 18p11.32, is a transmembrane scavenger receptor C-type lectin that recognizes certain bacteria and fungi, leading to phagocytosis (24). *COLEC12* has been shown to be aberrantly expressed and play an important role in a variety of cancers, Kong et al. found that the expression level of *COLEC12* was significantly elevated in a group of gastric cancer cells by controlled analysis; Sun et al. et al. found that *COLEC12* was highly expressed in patients with advanced gastric cancer and suggested that it has the ability to promote the proliferation, migration and invasion of gastric cancer cells and inhibit gastric cancer cell and inhibit apoptosis of gastric cancer cells; Wang et al. found that *COLEC12*, a cancer stemness-related gene, could predict the prognosis of colon adenocarcinoma patients (25–27). The results of this study revealed that *COLEC12* expression was down-regulated in SCLC. Moreover, the enrichment analysis revealed that it is involved in biological functions and pathways such as endocytic vesicle, scavenger receptor activity, and phagosome. The protein encoded by *COLEC12* is a scavenger receptor, which is expressed on various types of cells, like macrophages. It can recognize the microbial antigens of pathogens invading cells, initiate phagocytosis and activate downstream immune responses to combat and eliminate the pathogens (28). This is in line with the results of our enrichment analysis, underlining its crucial role in host defense. Based on these findings, we presume that a decrease in the expression of *COLEC12* may lead to a weakened ability to resist pathogens, subsequently influencing the development of SCLC. However, the research on the role of *COLEC12* in SCLC is relatively scarce at present. Our findings may offer a new perspective for understanding the cellular biology of SCLC.

*HP*, located on chromosomal locus 16q22.2, can encode a preproprotein, which is processed to produce haptoglobin. Haptoglobin is an acute phase response protein with anti-inflammatory and antioxidant properties, which is synthesized mainly by the liver, but can also be synthesized by the lungs, spleen, kidneys, skin and adipose tissue (29). The protein produced by the *HP* gene has been shown to play an important role in lung cancer. In NSCLC, several studies have found significantly elevated levels of haptoglobin in tumor tissues as well as in patient serum compared to normal controls, and suggested that high levels of haptoglobin correlate with TNM staging, lymph node metastasis, and distant metastasis (29, 30). In SCLC, Shah et al. found that the mean level of α- haptoglobin and β-haptoglobin in SCLC serum was increased (31). However, our study found that *HP* gene expression was down-regulated in SCLC tissues. The outcomes of GO enrichment analysis demonstrated its involvement in biological functions such as endocytic vesicle, endocytic vesicle lumen, haptoglobin-hemoglobin complex, tertiary granule lumen, specific granule lumen, and antioxidant activity. Free hemoglobin exhibits toxicity on account of nitric oxide scavenging and its direct pro-oxidative and pro-inflammatory activities. Previous research has indicated that the initial step in the classical pathway for the clearance of free hemoglobin hinges upon the formation of the hemoglobin-haptoglobin complex, and subsequently, the free hemoglobin is broken down within macrophages via endocytosis (32). This is consistent with the results of our enrichment analysis, indicating the importance of *HP* in the antioxidant and anti-inflammatory responses. We presume that the decreased expression of *HP* in tumor tissues may inhibit this regulatory pathway, thereby weakening the antioxidant and anti-inflammatory effects and promoting the development of SCLC. However, the research on the role of *HP* in SCLC is relatively scarce at present. Our findings may offer a new perspective for understanding the cellular biology of SCLC.

*PLLP* (Plasmolipin), located at chromosomal locus 16q13, is a component of synaptic plasma membranes, myelin sheaths and endocytic vesicles, and is involved in myelin formation (33, 34). This is consistent with the results of our enrichment analysis. Previous studies have found a significant increase in *PLLP* expression in mouse models of breast cancer and melanoma brain metastasis, and speculated that it may play a role in brain metastasis of these two malignant tumors (35, 36). In glioblastoma, Luo et al. found that the expression of *PLLP* in the cells of peritumoral brain zone was lower than that in the cells of the core zone of the tumor (37). Recent study have reported a significant correlation between *PLLP* and renal smoky cell carcinoma, renal clear cell carcinoma, and endometrial carcinoma of the uterine corpus (34). In this study, we found that the expression of *PLLP* is down-regulated in small cell lung cancer and the results of enrichment analysis indicated its involvement in biological functions such as compact myelin, myelin sheath, and structural constituent of myelin sheath. As the main component of myelin, *PLLP* promotes myelin formation by inducing myelin precursor domains in the Golgi apparatus after phosphorylation (38).Based on our findings, we speculate that the reason why the decreased expression of *PLLP* can promote the development of SCLC might be related to the neuroendocrine characteristics of SCLC. However, there are limited studies related to the role of *PLLP* in SCLC and further studies are still needed to explore its specific mechanism of action in SCLC.

In addition to exploring gene-specific effects on SCLC, we expanded our study to the immunological level of SCLC. We performed an analysis of immune cell infiltration levels by the CIBERSORT algorithm. We found significantly different immune cell infiltration levels between SCLC and normal tissue samples, which is broadly in line with previous findings (39, 40). Based on the results of differential expression analysis, we further analyzed the correlations between the five co-expressed genes and 22 immune cell subpopulations, and the results showed that all five co-expressed genes were correlated with some immune cells, especially *PSRC1* and *COLEC12*. Specifically, we found that in the up-regulated cancer-enriched genes (CEGs), *PSRC1* was positively correlated with T cells follicular helper and activated NK cells. Moreover, in the down-regulated CEGs, *COLEC12* was negatively correlated with T cells follicular helper. Compared with the control group, the proportion of T cells follicular helper in SCLC was significantly increased. Therefore we further explored the activity levels of *PSRC1* and *COLEC12* in SCLC by GSEA method, and the results showed that *PSRC1* and *COLEC12* play a multifaceted and complex role in the pathogenesis of SCLC. The expression levels of *PSRC1* may be associated with changes in biological functional activities related to immune responses, chromosome segregation, and others. The active pathways in the *PSRC1* high-expression group indicated the possible presence of cell division process which is mediated by the regulatory role of *PSRC1* in SCLC, and the active pathways in the *PSRC1* low-expression group indicated the possible presence of humoral immune responses and the metabolic processes of cytochrome P450 which are mediated by the regulatory role of *PSRC1* in SCLC. While the expression levels of *COLEC12* may be associated with changes in biological functional activities related to cell cycle, immune response, and others. The active pathways in the *COLEC12* high-expression group indicated the possible presence of immune response which is mediated by the regulatory role of *COLEC12* in SCLC, and the active pathways in the *COLEC12* low-expression group indicated the possible presence of process of cell division which is mediated by the regulatory role of *COLEC12* in SCLC.

**Figure 12:**
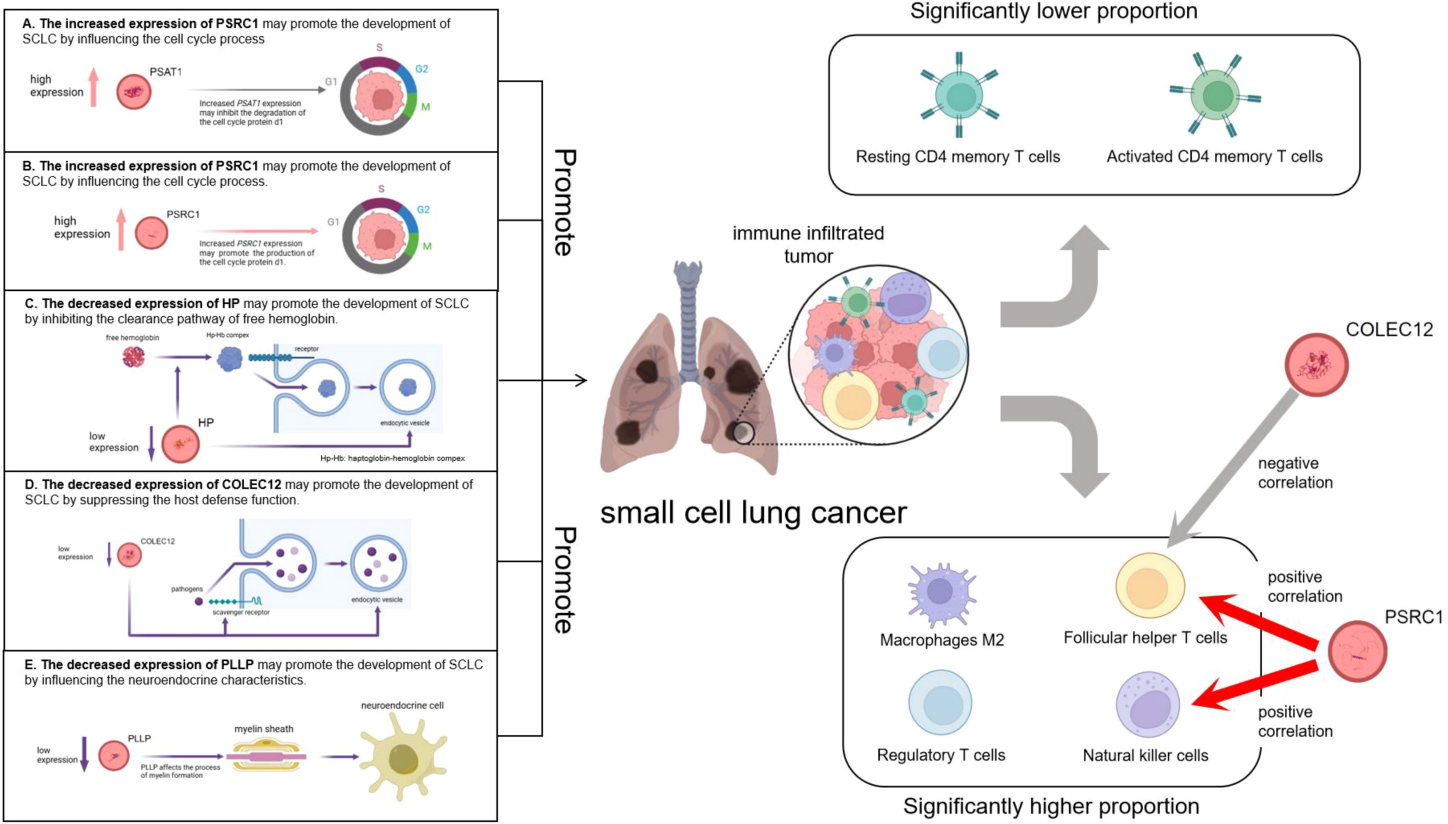
Graphic abstract. The left part illustrates the pathogenesis model of small cell lung cancer suggested by five co-expressed genes. The right part shows the infiltration of immune cells in small cell lung cancer as well as the relationships between PSRC1, COLEC12 and the infiltration of immune cells.

Finally, we used a validation group for difference expression analysis, and the results were consistent with those of the MR analysis, which confirmed the reliability of our findings. The elevated expression of *PSRC1* may promote the development of SCLC by influencing the cell cycle process and immune cell infiltration, while the increased expression of *PSAT1* may contribute to the development of SCLC by affecting the cell cycle process. The decreased expression of COLEC12 may suppress the host defense function and immune cell infiltration, thereby promoting the development of SCLC; The decreased expression of HP may inhibit the clearance pathway of free hemoglobin, leading to the enhancement of oxidative stress and inflammatory response, thereby promoting the development of SCLC; and the decreased expression of *PLLP* may promote the development of SCLC by influencing the neuroendocrine characteristics. A graphical summary is visible in **Figure 12**. However, all these possibilities need to be verified by further experiments to elucidate the specific roles of these genes in SCLC. Certainly, the potential therapeutic targets identified in the latest research merit further attention. For instance, the research team found that the expression of Three prime repair exonuclease 1 (TREX1) is up-regulated in SCLC. Through fundamental experiments, it was discovered that suppressing its expression can enhance anti-tumor immunity and augment the efficacy of chemotherapy and/or immunotherapy for chemotherapy-resistant SCLC (41). The other research team analyzed the genome-wide loss-of-function screening database to look for vulnerabilities in SCLC and they believed that S-phase kinase-associated protein 2 (*SKP2)* could be regarded as a new therapeutic target for SCLC, regardless of whether it is RB1 wild-type or mutant SCLC (42).

In summary, this study performed a detailed analysis of SCLC using bioinformatics as well as statistical methods to identify key genes and pathways, focusing on the association between co-expressed genes (*PSAT1, PSRC1, COLEC12, PLLP, and HP*) and SCLC. These genes and their associated functions and pathways may be potential targets for targeting specific molecular pathways for the treatment of SCLC, which lays a theoretical foundation for the subsequent development of new therapeutic interventions. There are some limitations in this study. First, only a single bioinformatics analysis was performed, lack of biological experiments for validation. Second, due to the incomplete information in the original datasets, at present, this study has not yet carried out hierarchical analyses on factors such as age, gender, and disease stage. In the future, we will conduct deeper clinical studies and laboratory validation.

## Data Availability

The original contributions presented in the study are included in the article/**Supplementary Material**. Further inquiries can be directed to the corresponding author/s.
