# Supplementary material for: Exploring potential therapeutic targets for small cell lung cancer based on transcriptomics combined with Mendelian randomization analysis
Source: Front Immunol. 2025 Jan 13;15:1464259. doi: 10.3389/fimmu.2024.1464259 (PMC11769988; doi:10.3389/fimmu.2024.1464259)
Supplement: Supplementary file 1 [file Image1.pdf]

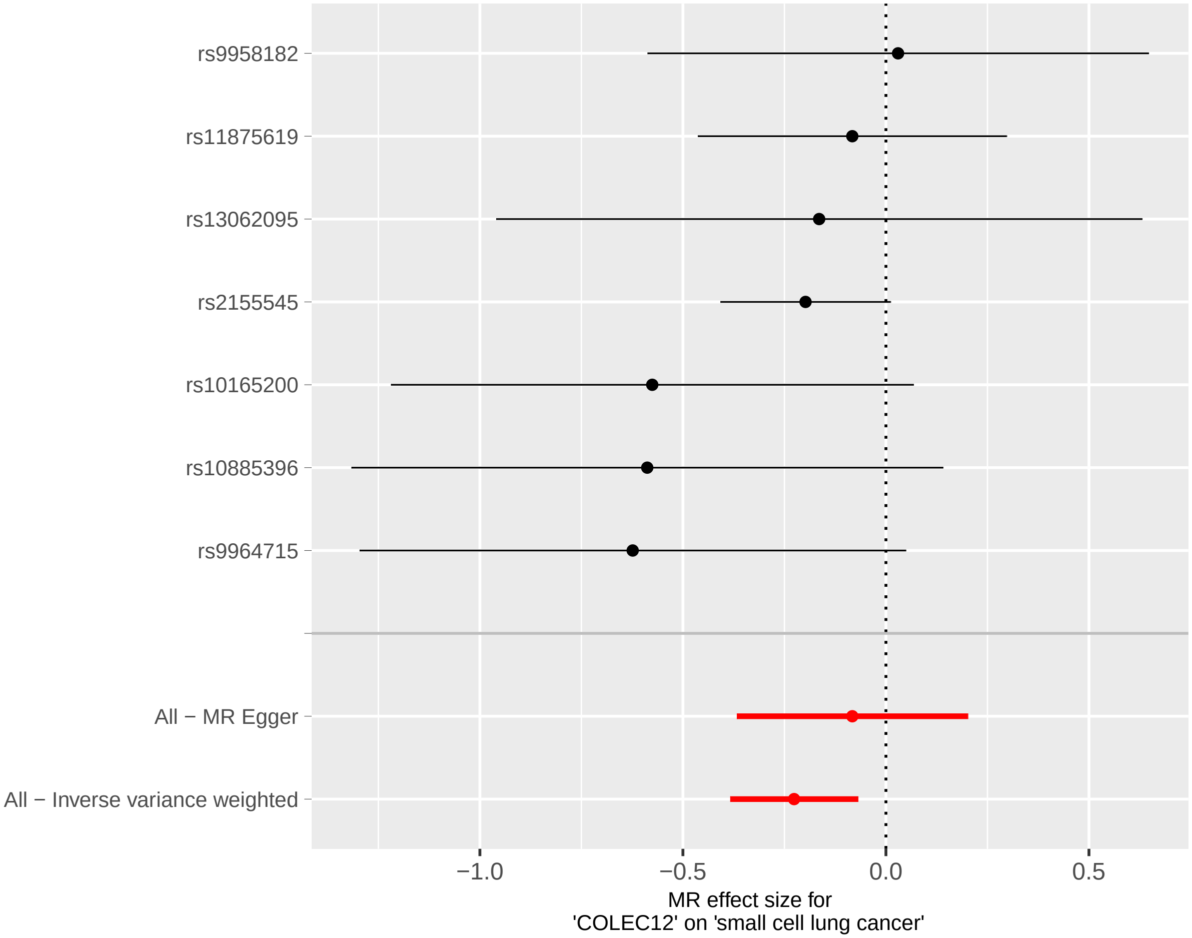

rs9958182
rs11875619
rs13062095
rs2155545
rs10165200
rs10885396
rs9964715
All − MR Egger
All − Inverse variance weighted
−1.0
−0.5
0.0
0.5
MR effect size for
'COLEC12' on 'small cell lung cancer'

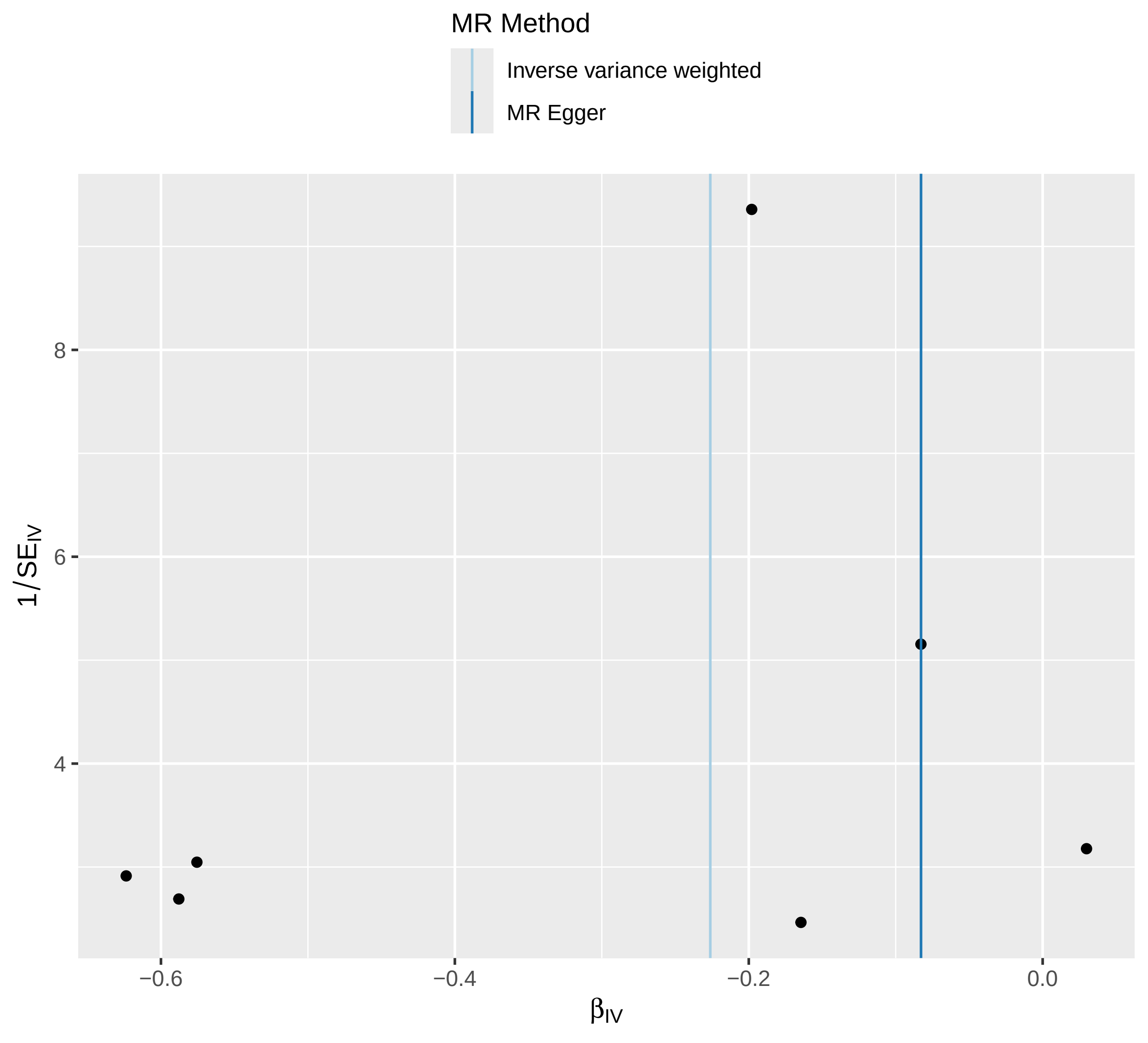
MR Method
Inverse variance weighted
MR Egger
8
6
4
1/SE_IV
−0.6
−0.4
−0.2
0.0
β_IV

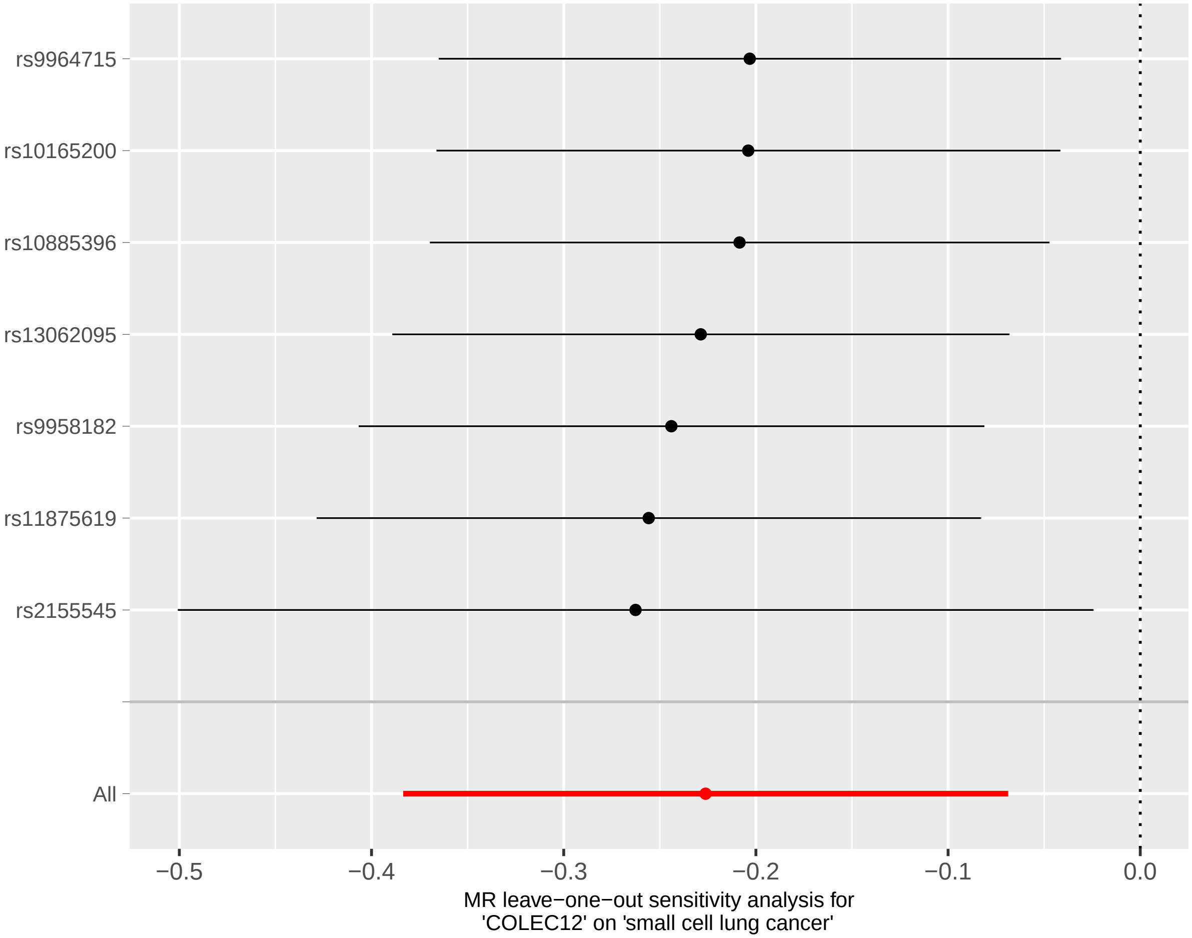
rs9964715
rs10165200
rs10885396
rs13062095
rs9958182
rs11875619
rs2155545
All
−0.5
−0.4
−0.3
−0.2
−0.1
0.0
MR leave−one−out sensitivity analysis for
'COLEC12' on 'small cell lung cancer'

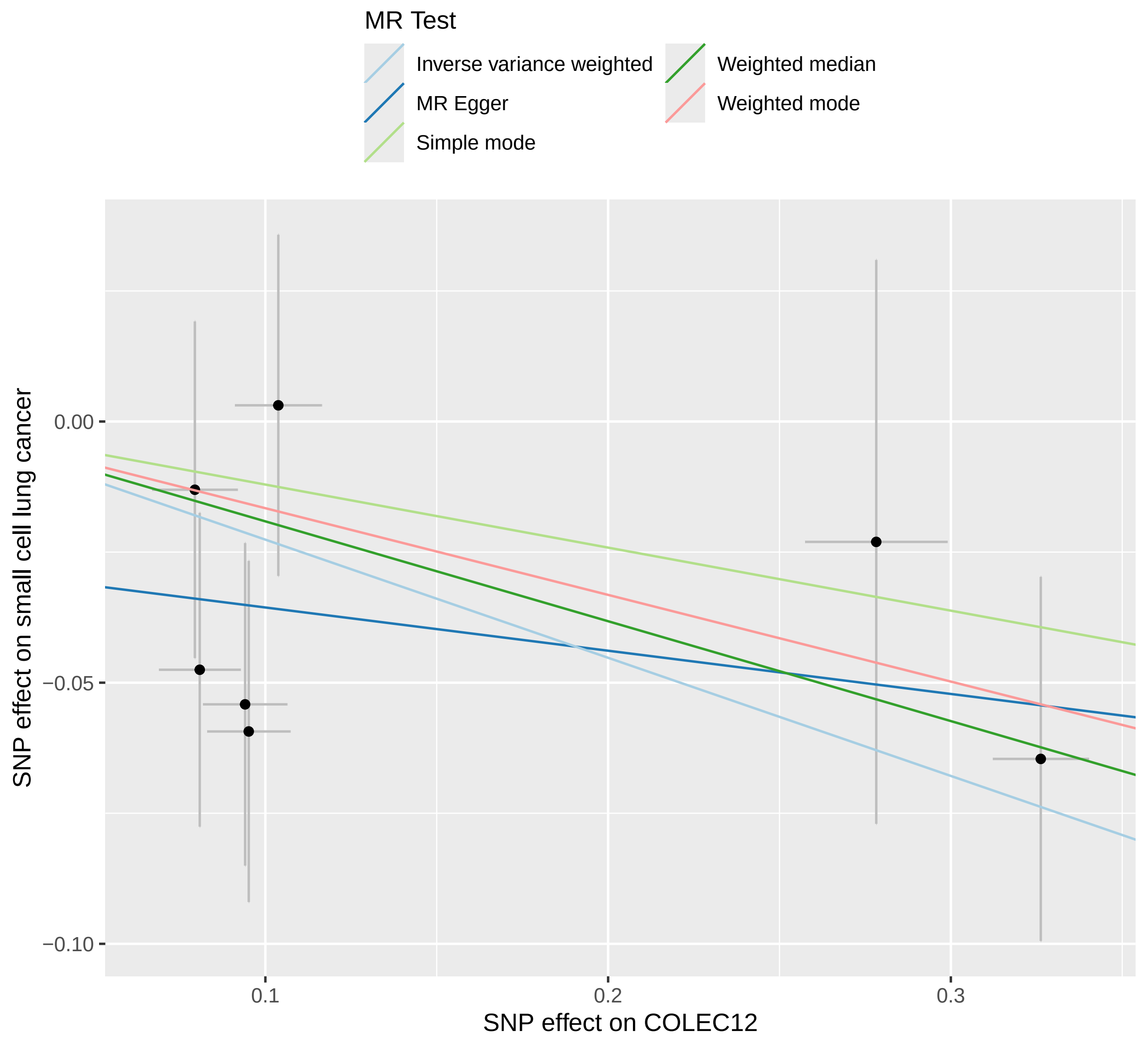

MR Test
Inverse variance weighted
MR Egger
Simple mode
Weighted median
Weighted mode
0.00
−0.05
−0.10
0.1
0.2
0.3
SNP effect on small cell lung cancer
SNP effect on COLEC12

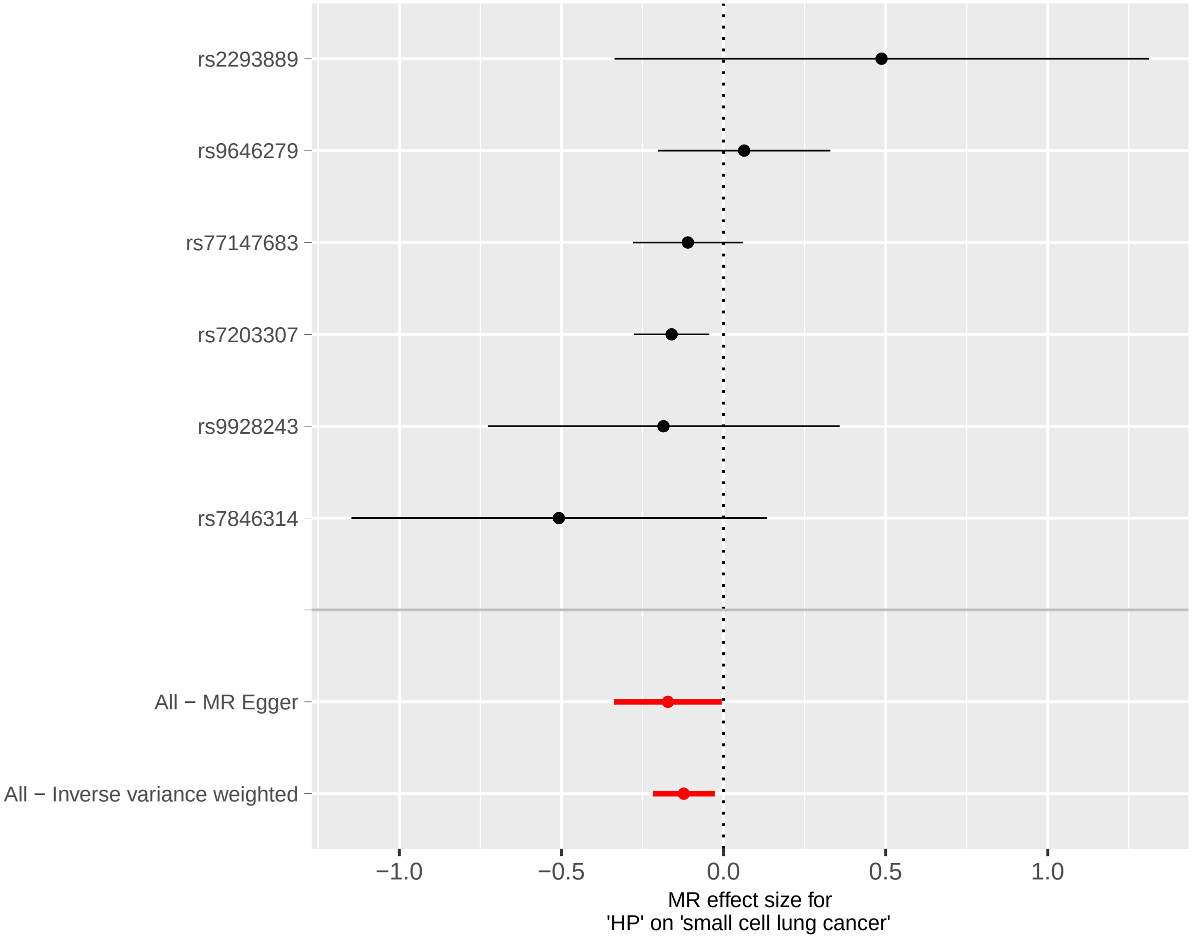
rs2293889
rs9646279
rs77147683
rs7203307
rs9928243
rs7846314
All − MR Egger
All − Inverse variance weighted
−1.0
−0.5
0.0
0.5
1.0
MR effect size for
'HP' on 'small cell lung cancer'

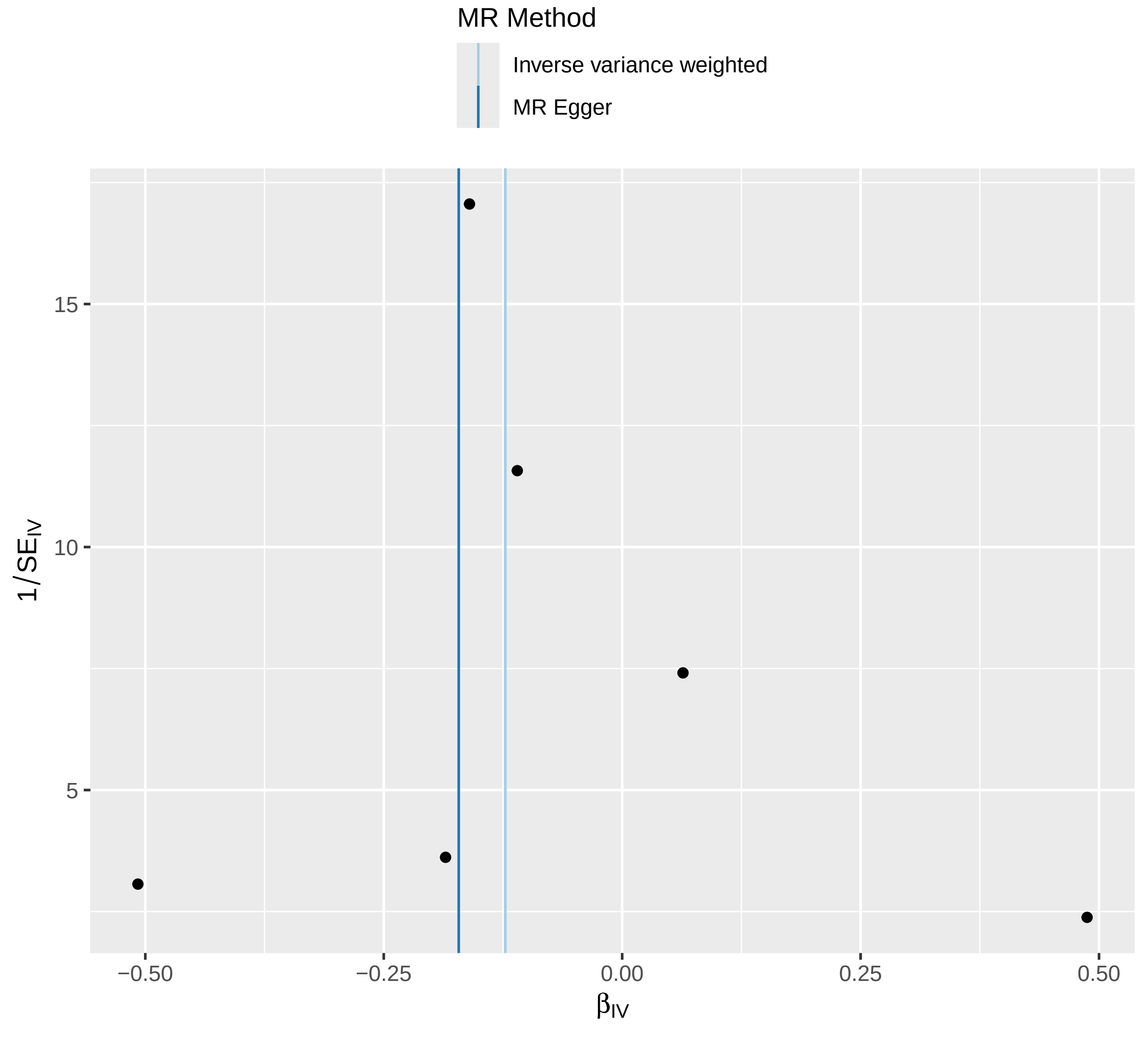
MR Method
Inverse variance weighted
MR Egger
15
10
5
1/SE_IV
−0.50
−0.25
0.00
0.25
0.50
β_IV

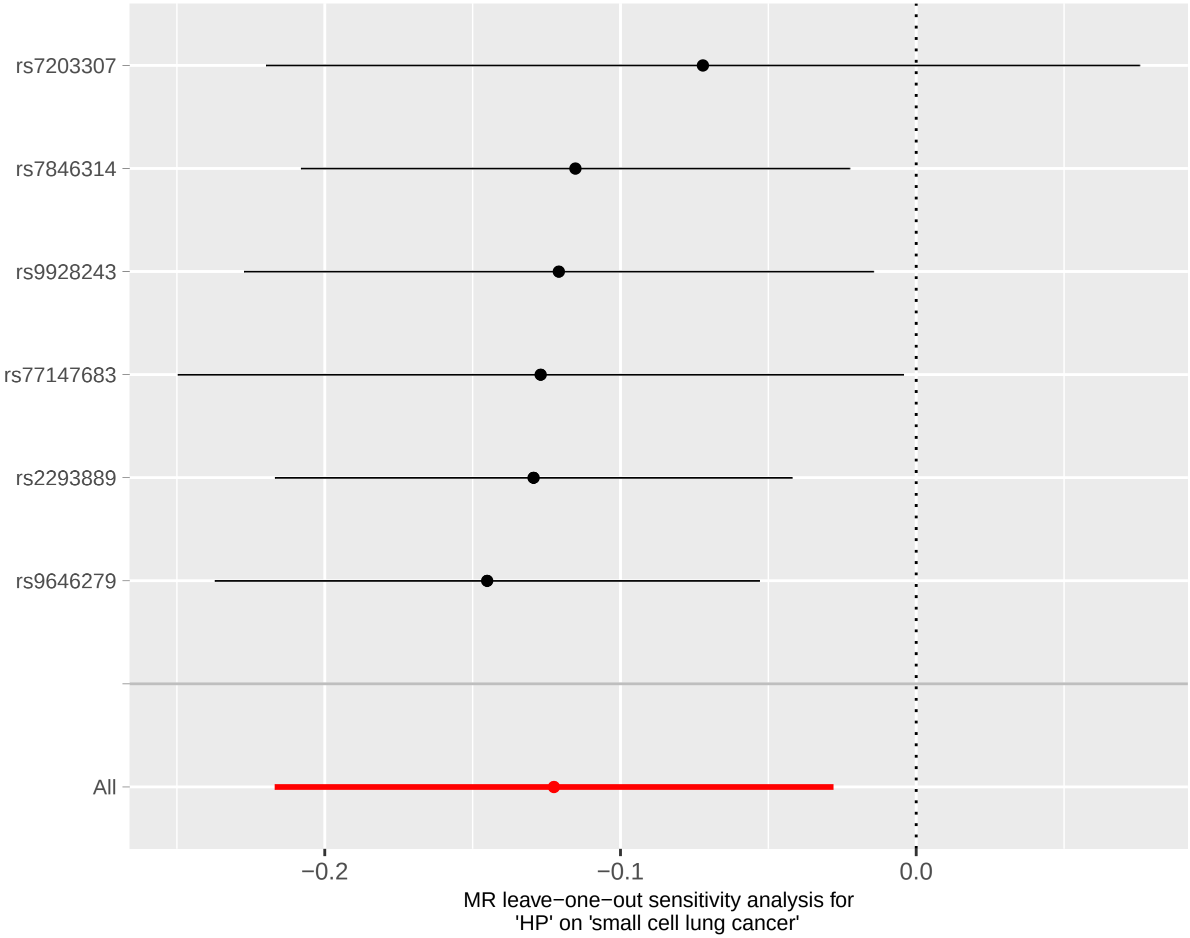

rs7203307
rs7846314
rs9928243
rs77147683
rs2293889
rs9646279
All
−0.2
−0.1
0.0
MR leave−one−out sensitivity analysis for
'HP' on 'small cell lung cancer'

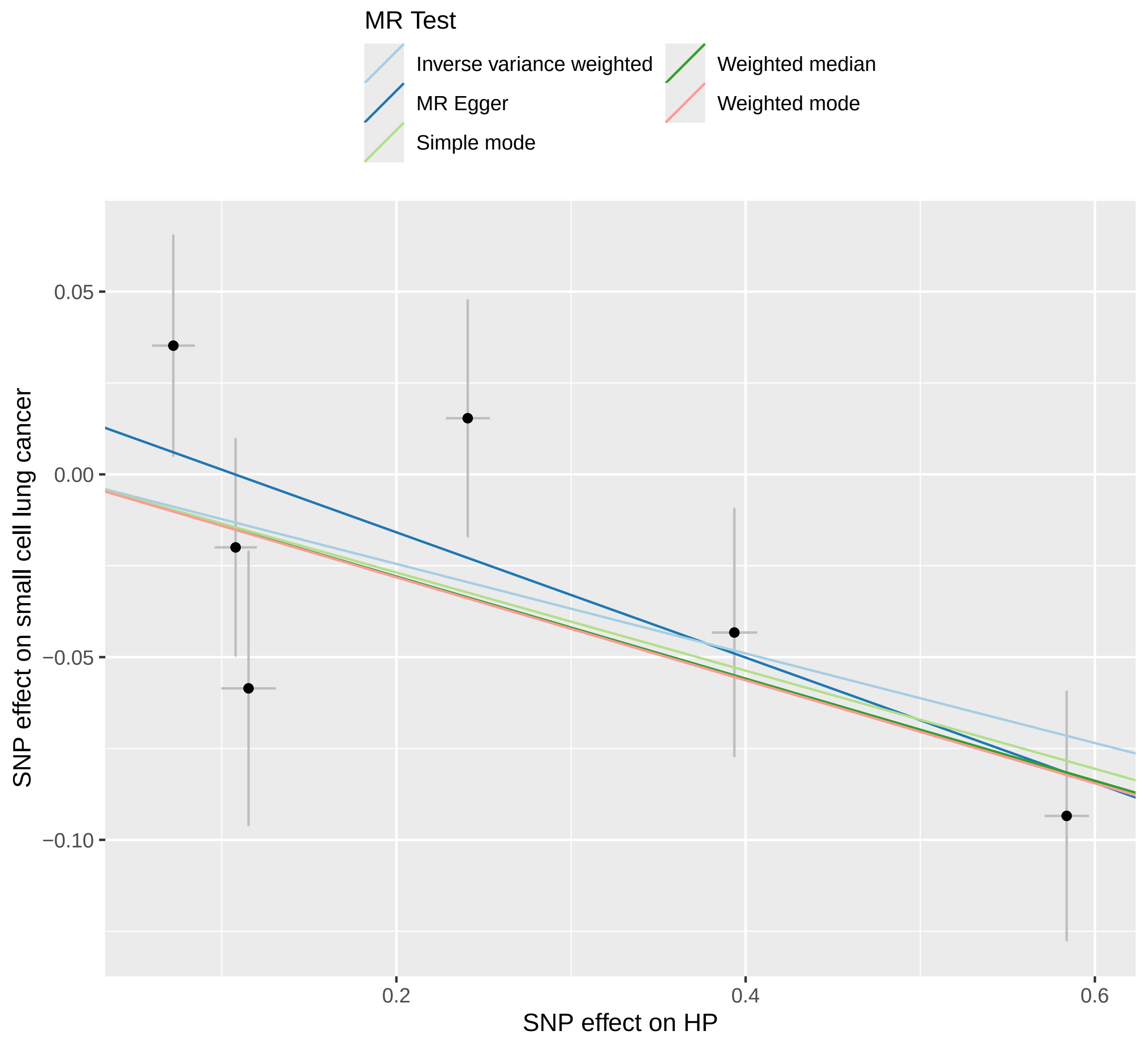
MR Test
Inverse variance weighted
MR Egger
Simple mode
Weighted median
Weighted mode
0.05
0.00
−0.05
−0.10
0.2
0.4
0.6
SNP effect on small cell lung cancer
SNP effect on HP

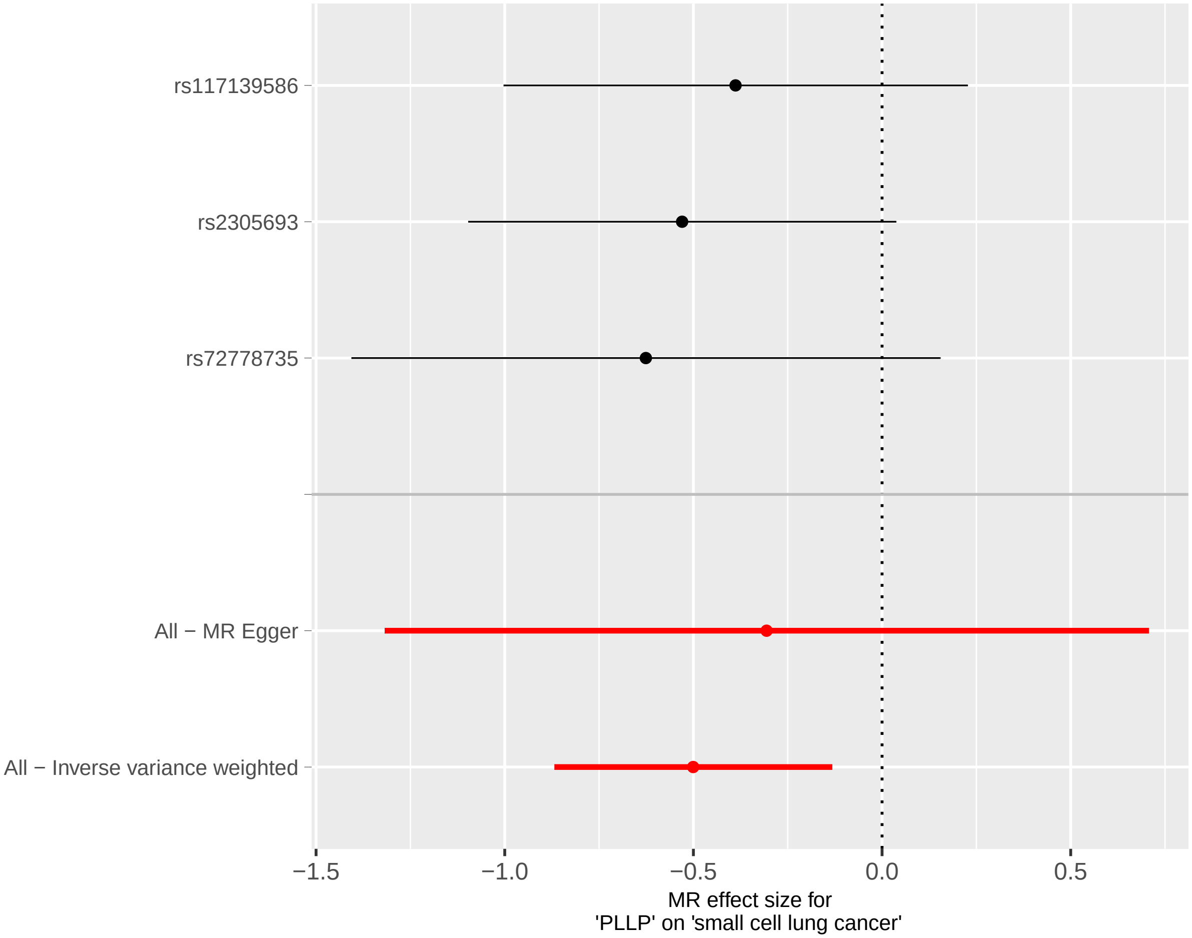

rs117139586
rs2305693
rs72778735
All − MR Egger
All − Inverse variance weighted
−1.5
−1.0
−0.5
0.0
0.5
MR effect size for
'PLLP' on 'small cell lung cancer'

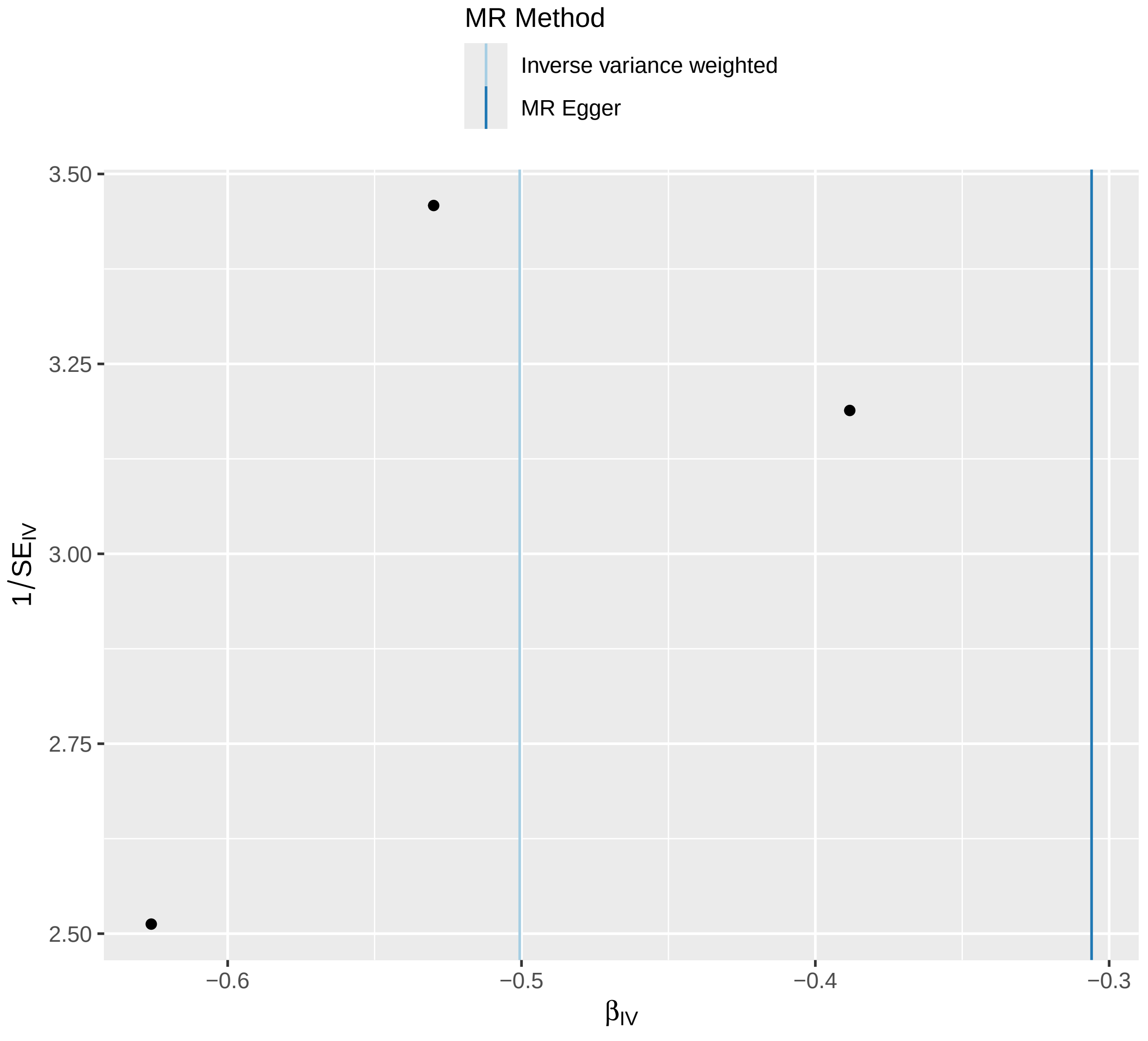
MR Method
Inverse variance weighted
MR Egger
3.50
3.25
3.00
2.75
2.50
1/$SE_{IV}$
−0.6
−0.5
−0.4
−0.3
$\beta_{IV}$

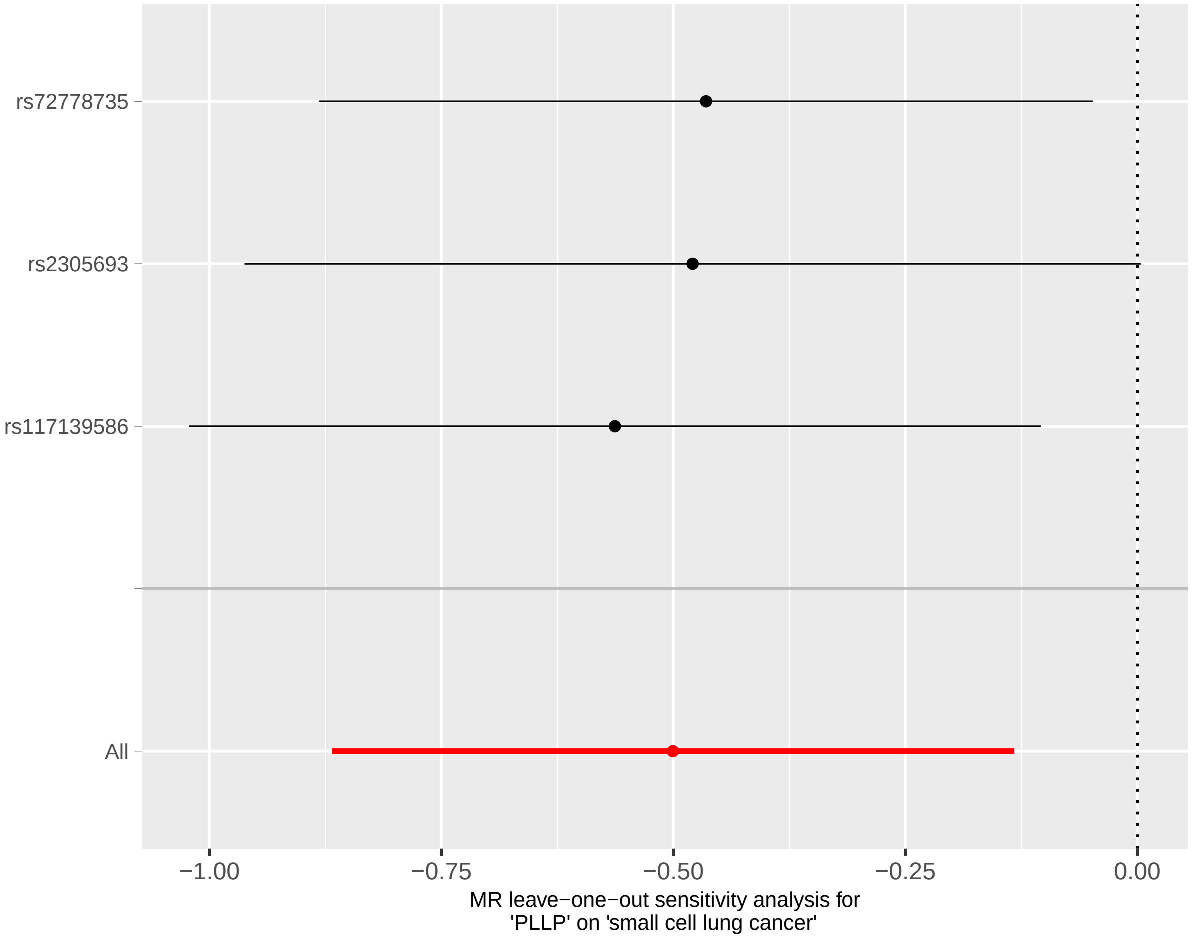

rs72778735
rs2305693
rs117139586
All
−1.00
−0.75
−0.50
−0.25
0.00
MR leave−one−out sensitivity analysis for
'PLLP' on 'small cell lung cancer'

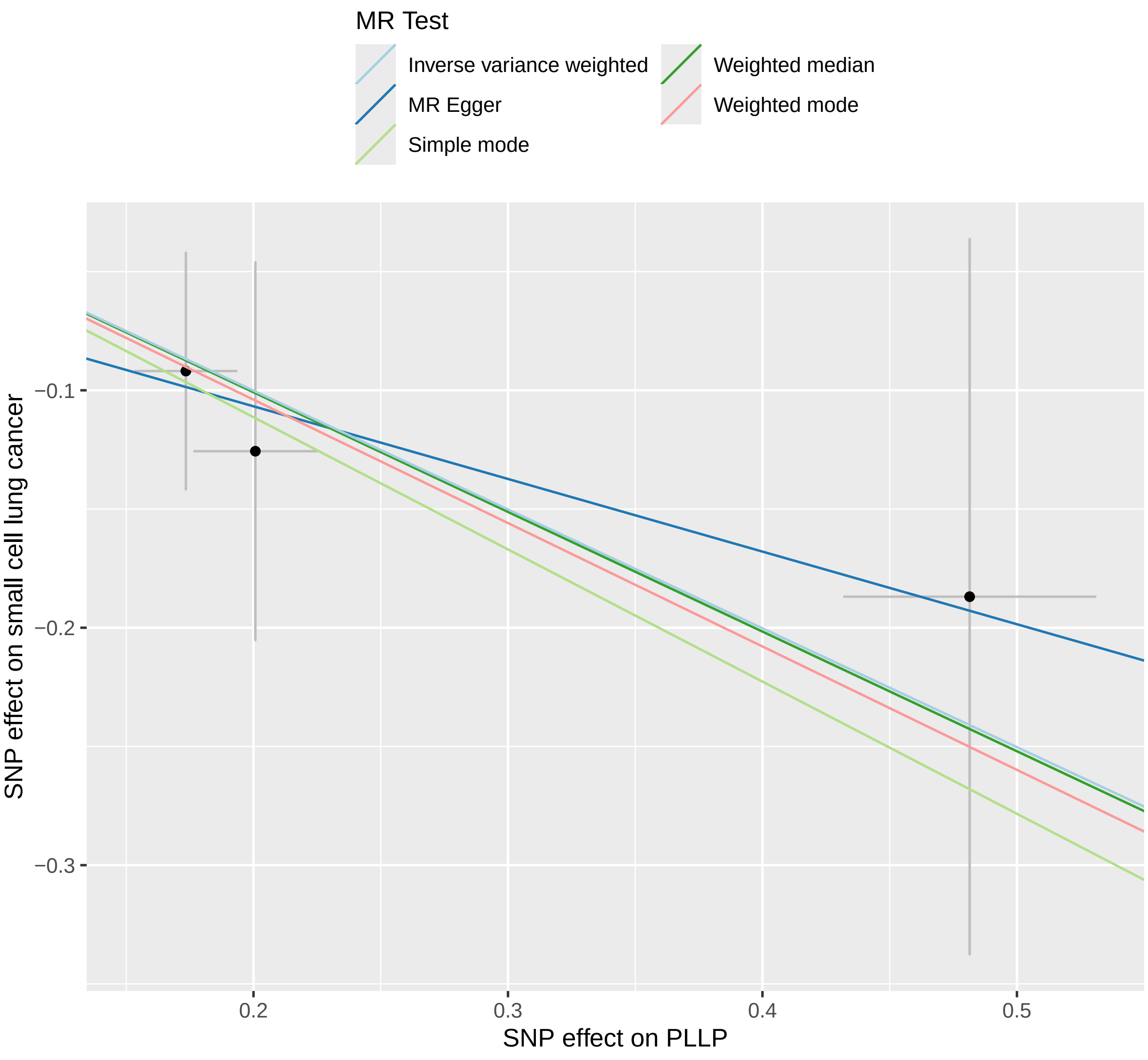
MR Test
Inverse variance weighted
MR Egger
Simple mode
Weighted median
Weighted mode
−0.1
−0.2
−0.3
0.2
0.3
0.4
0.5
SNP effect on small cell lung cancer
SNP effect on PLLP

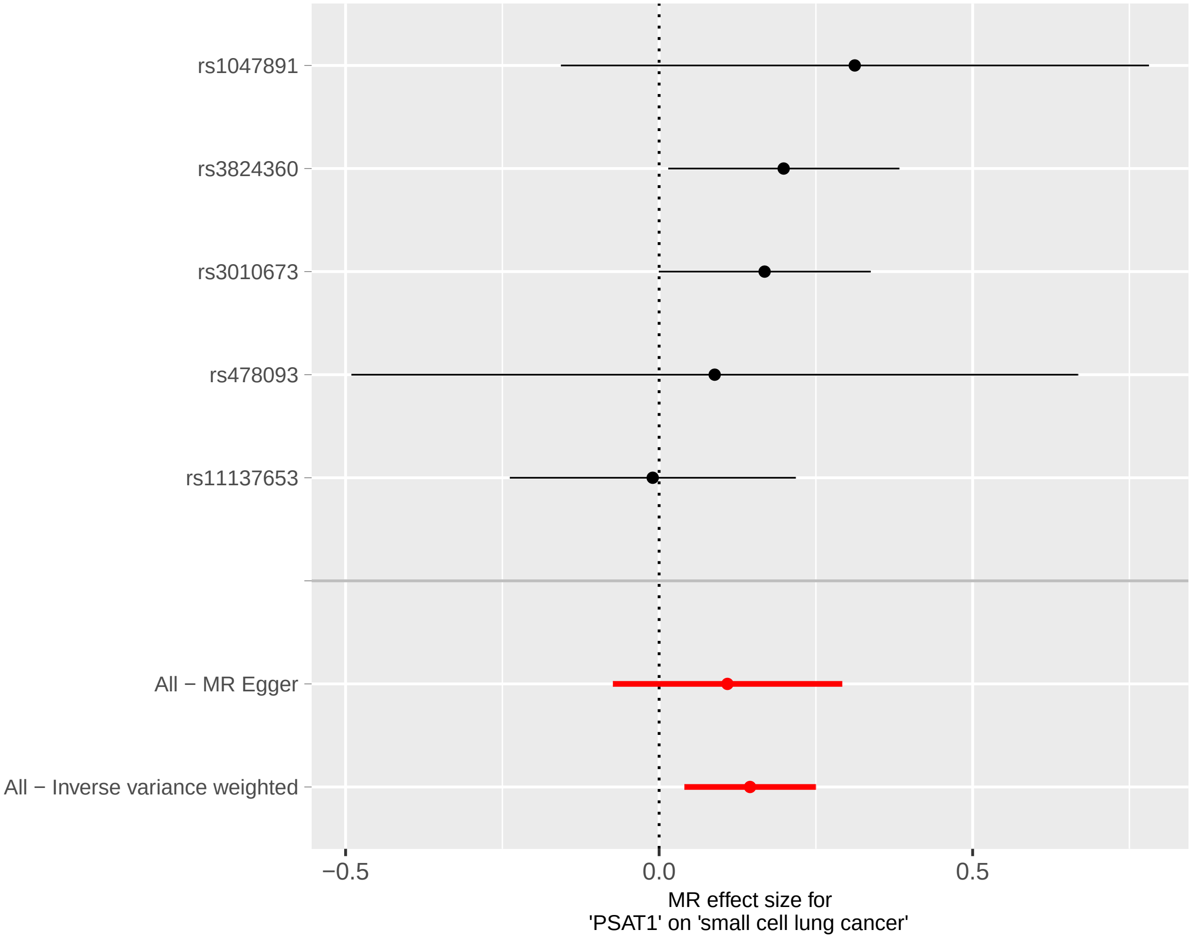
rs1047891
rs3824360
rs3010673
rs478093
rs11137653
All − MR Egger
All − Inverse variance weighted
−0.5
0.0
0.5
MR effect size for
'PSAT1' on 'small cell lung cancer'

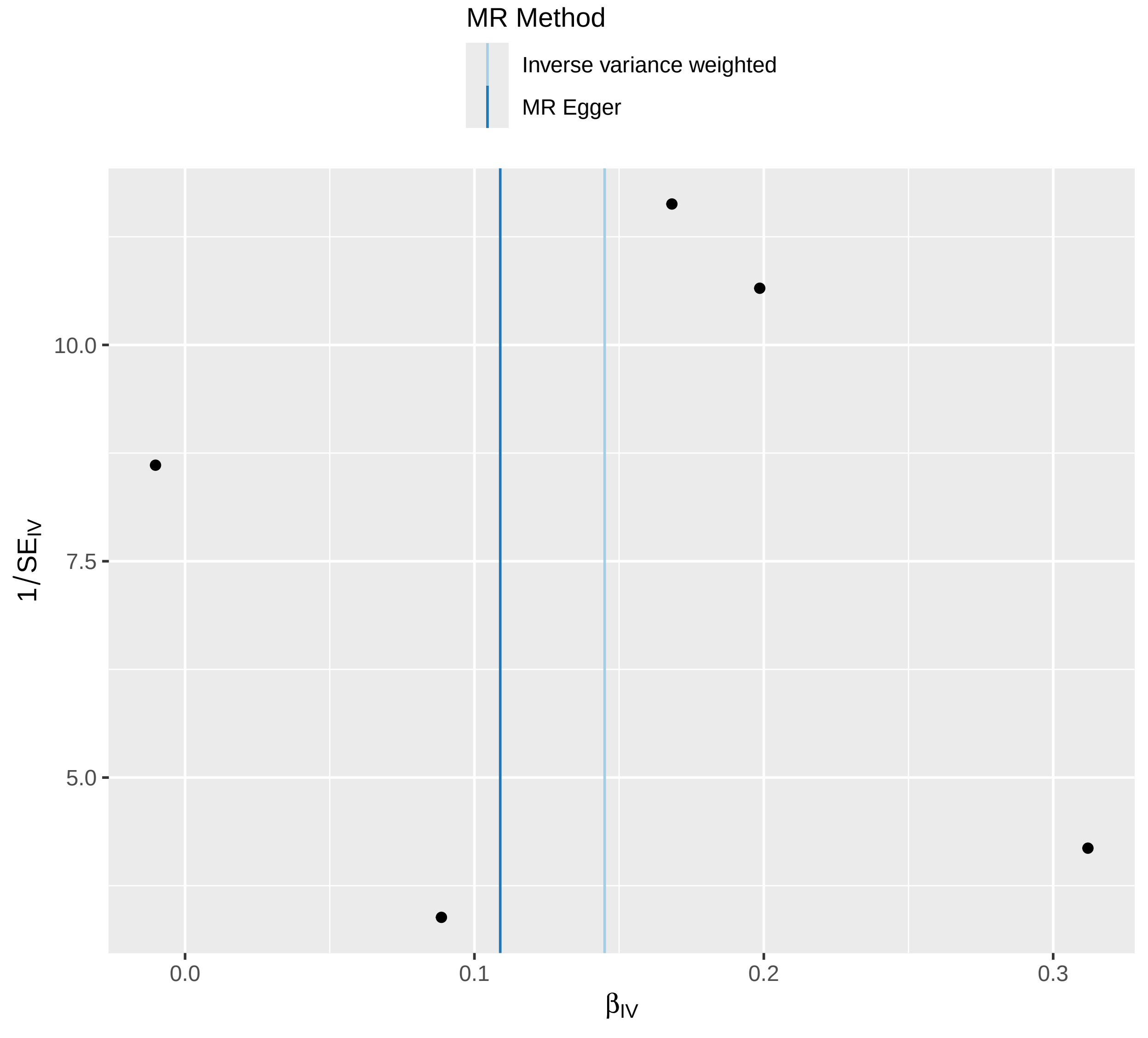

MR Method
Inverse variance weighted
MR Egger
10.0
7.5
5.0
0.0
0.1
0.2
0.3
$1/SE_{IV}$
$\beta_{IV}$

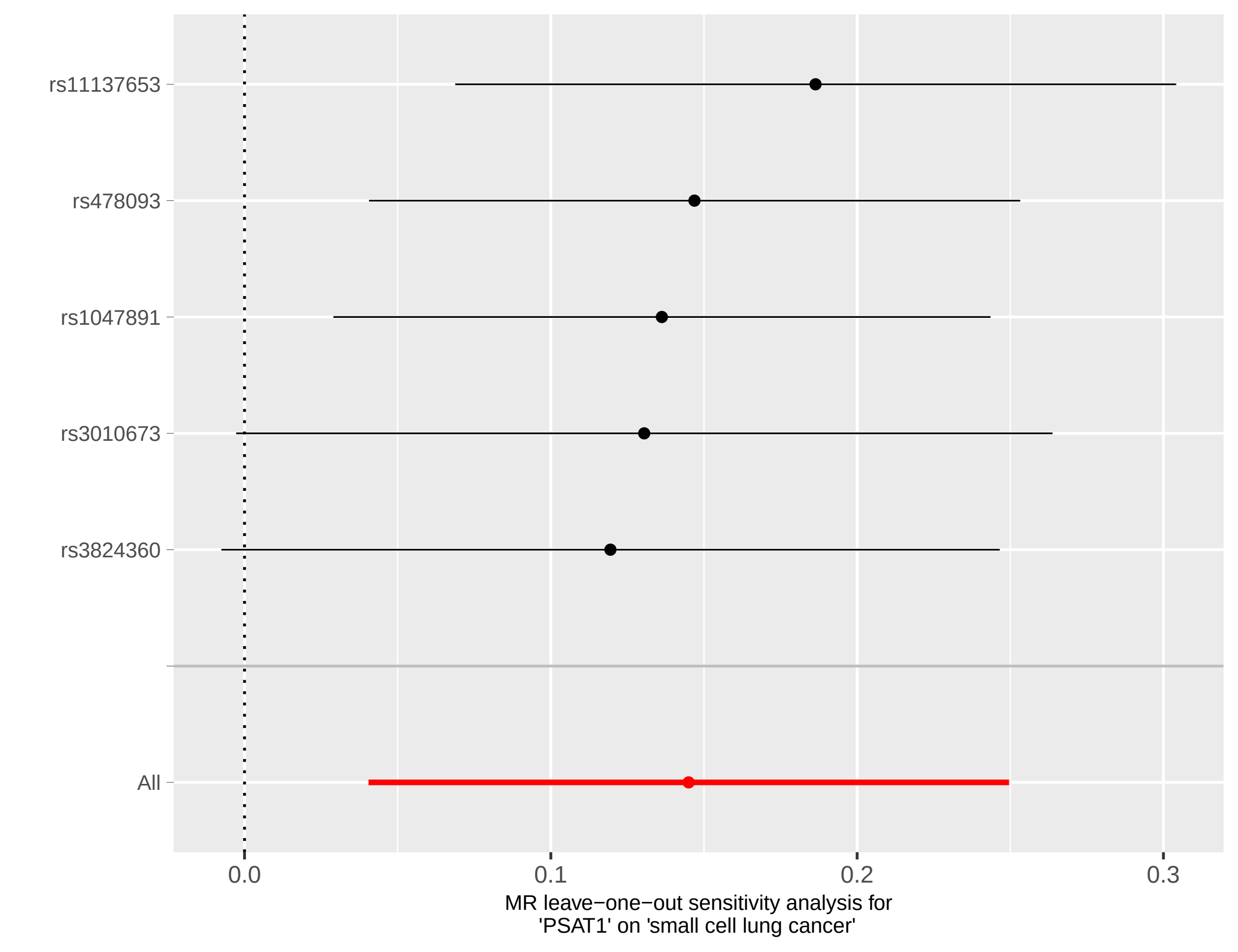

rs11137653
rs478093
rs1047891
rs3010673
rs3824360
All
0.0
0.1
0.2
0.3
MR leave−one−out sensitivity analysis for
'PSAT1' on 'small cell lung cancer'

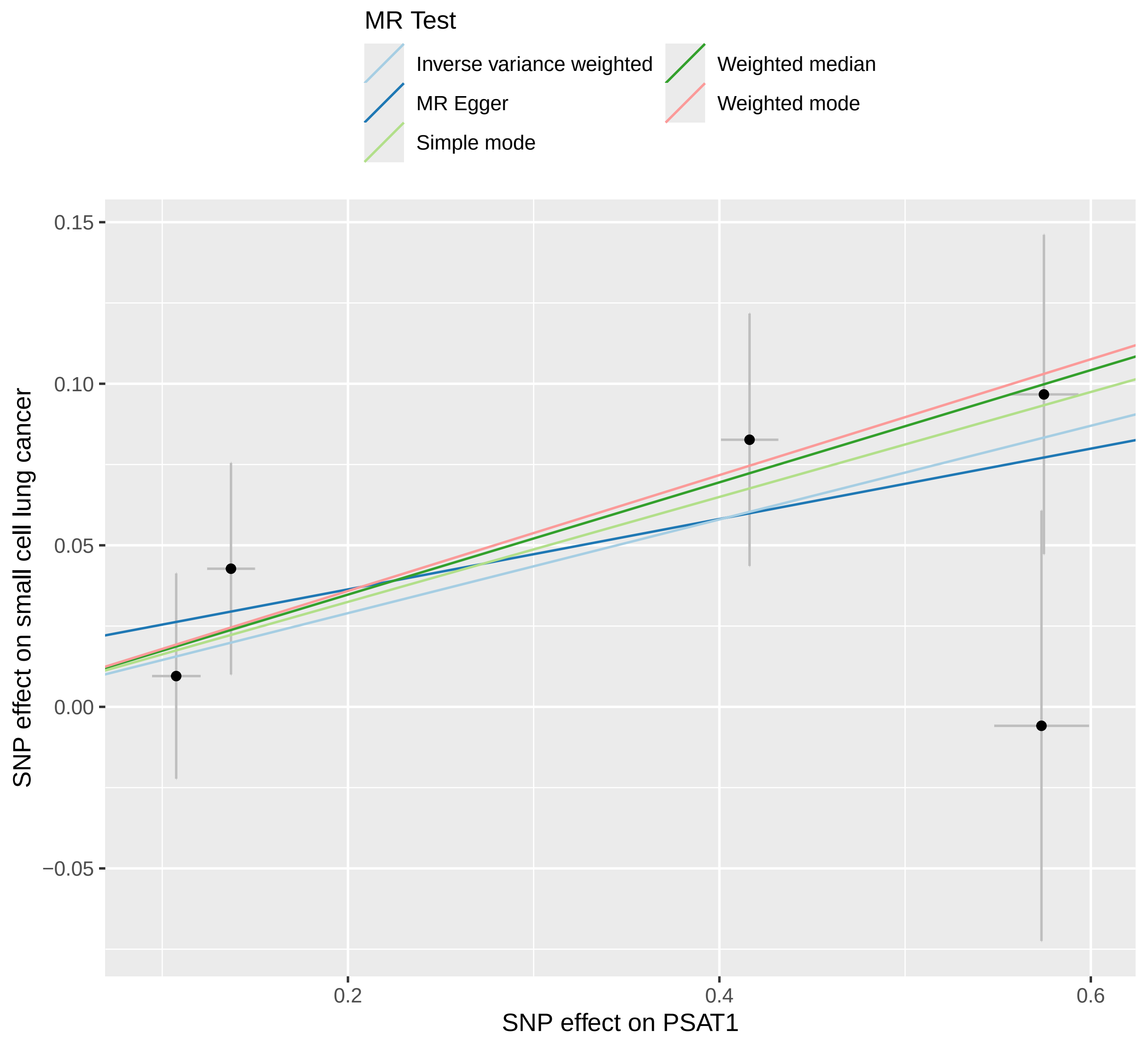
MR Test
Inverse variance weighted
MR Egger
Simple mode
Weighted median
Weighted mode
SNP effect on small cell lung cancer
0.15
0.10
0.05
0.00
−0.05
0.2
0.4
0.6
SNP effect on PSAT1

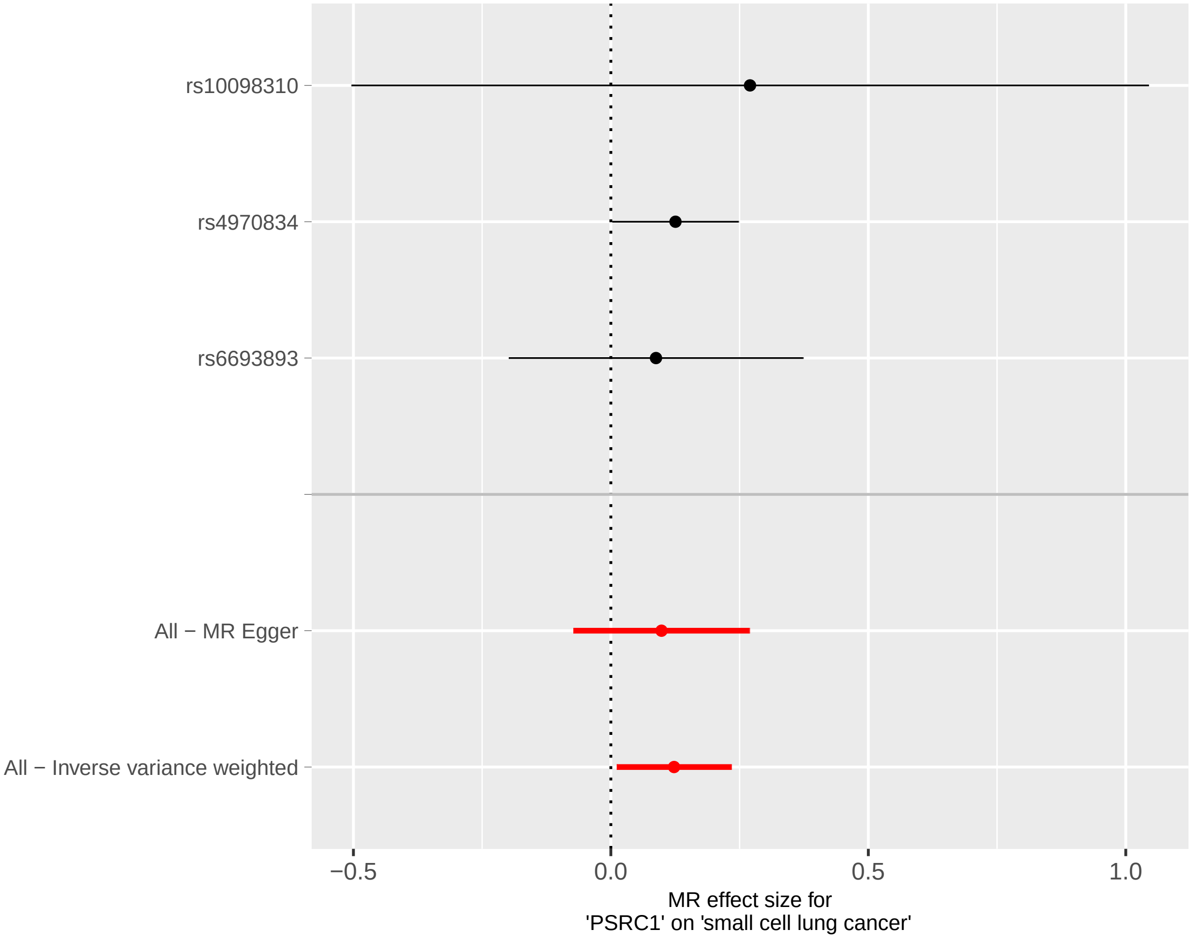
rs10098310
rs4970834
rs6693893
All − MR Egger
All − Inverse variance weighted
−0.5
0.0
0.5
1.0
MR effect size for
'PSRC1' on 'small cell lung cancer'

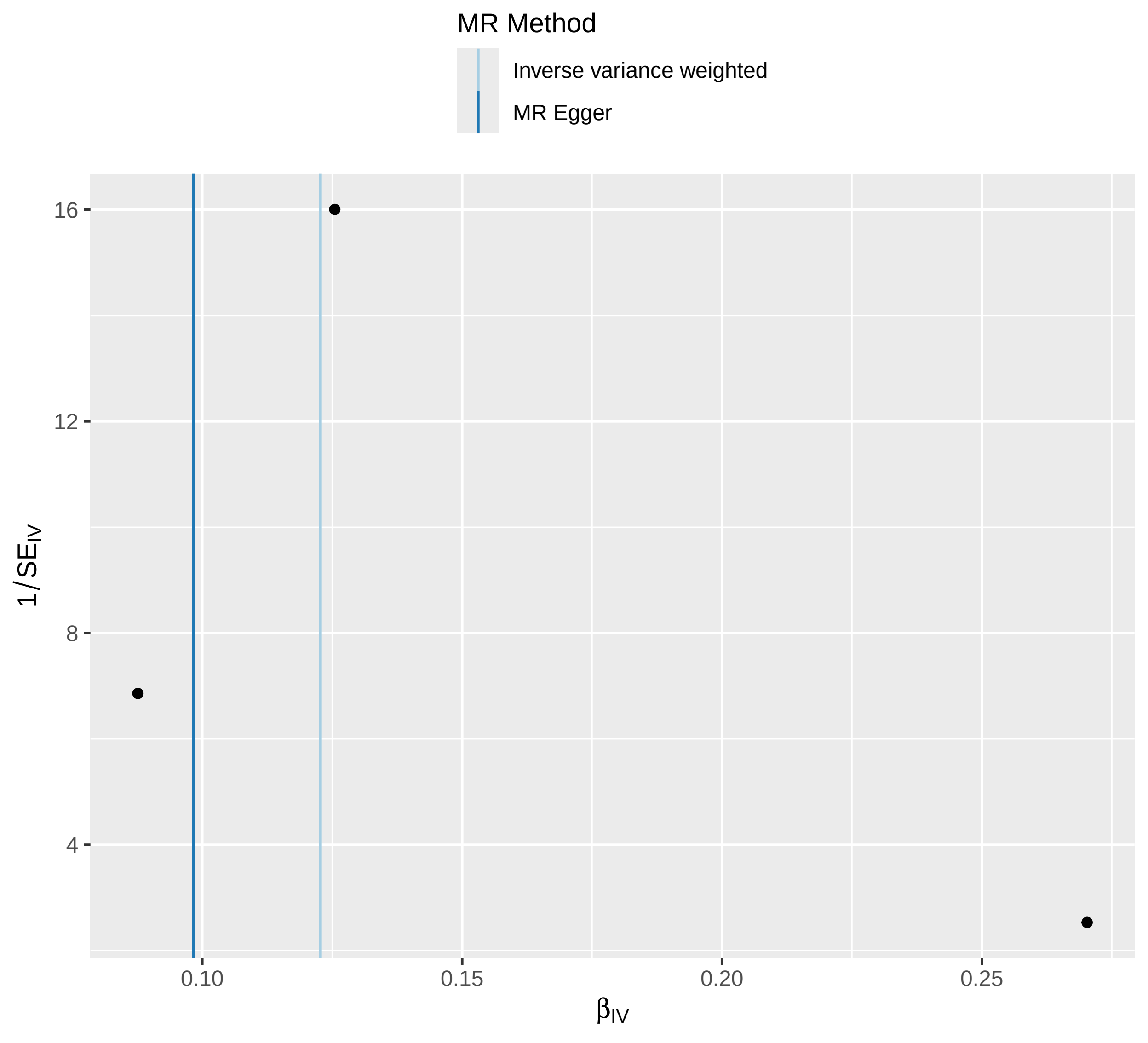
MR Method
Inverse variance weighted
MR Egger
16
12
8
4
1/SE_IV
0.10
0.15
0.20
0.25
β_IV

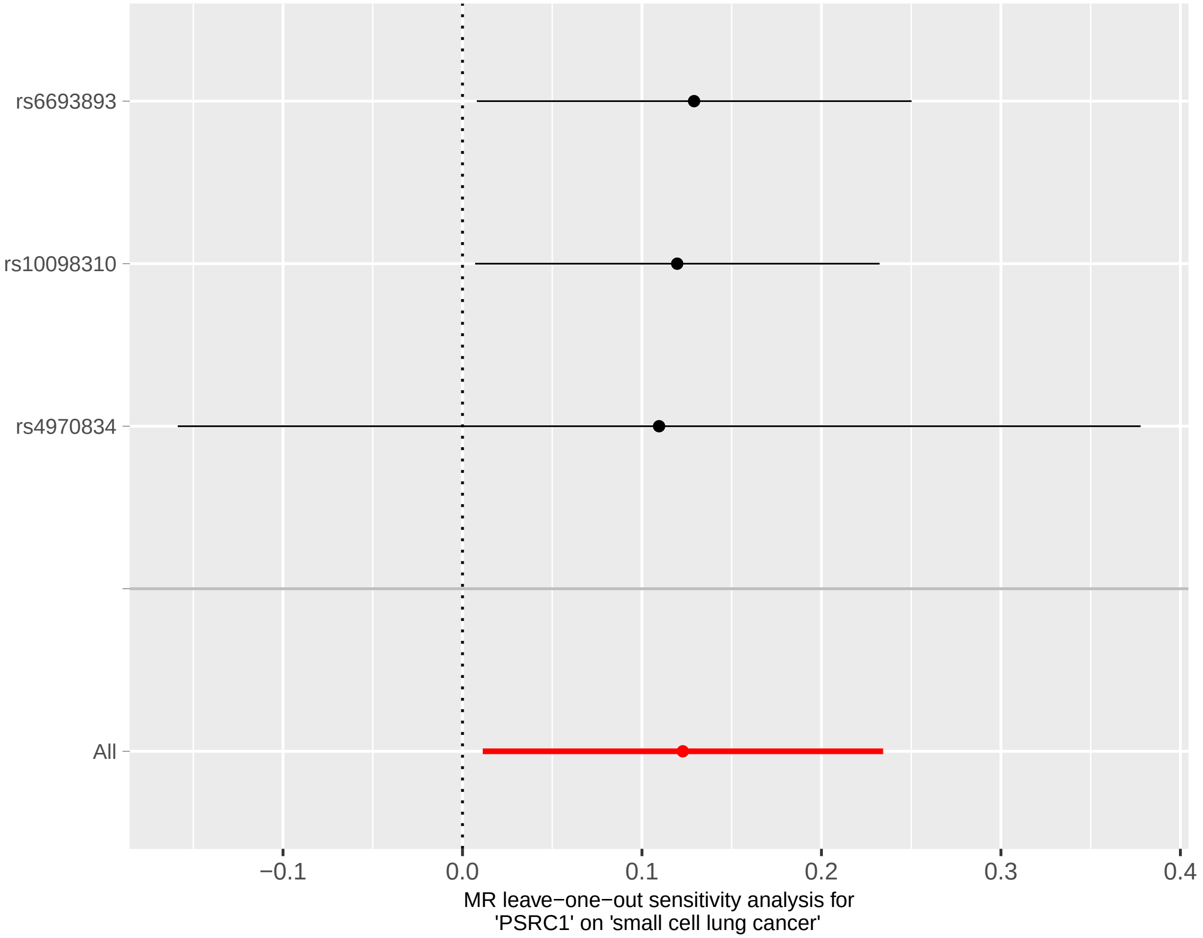

rs6693893
rs10098310
rs4970834
All
−0.1
0.0
0.1
0.2
0.3
0.4
MR leave−one−out sensitivity analysis for
'PSRC1' on 'small cell lung cancer'

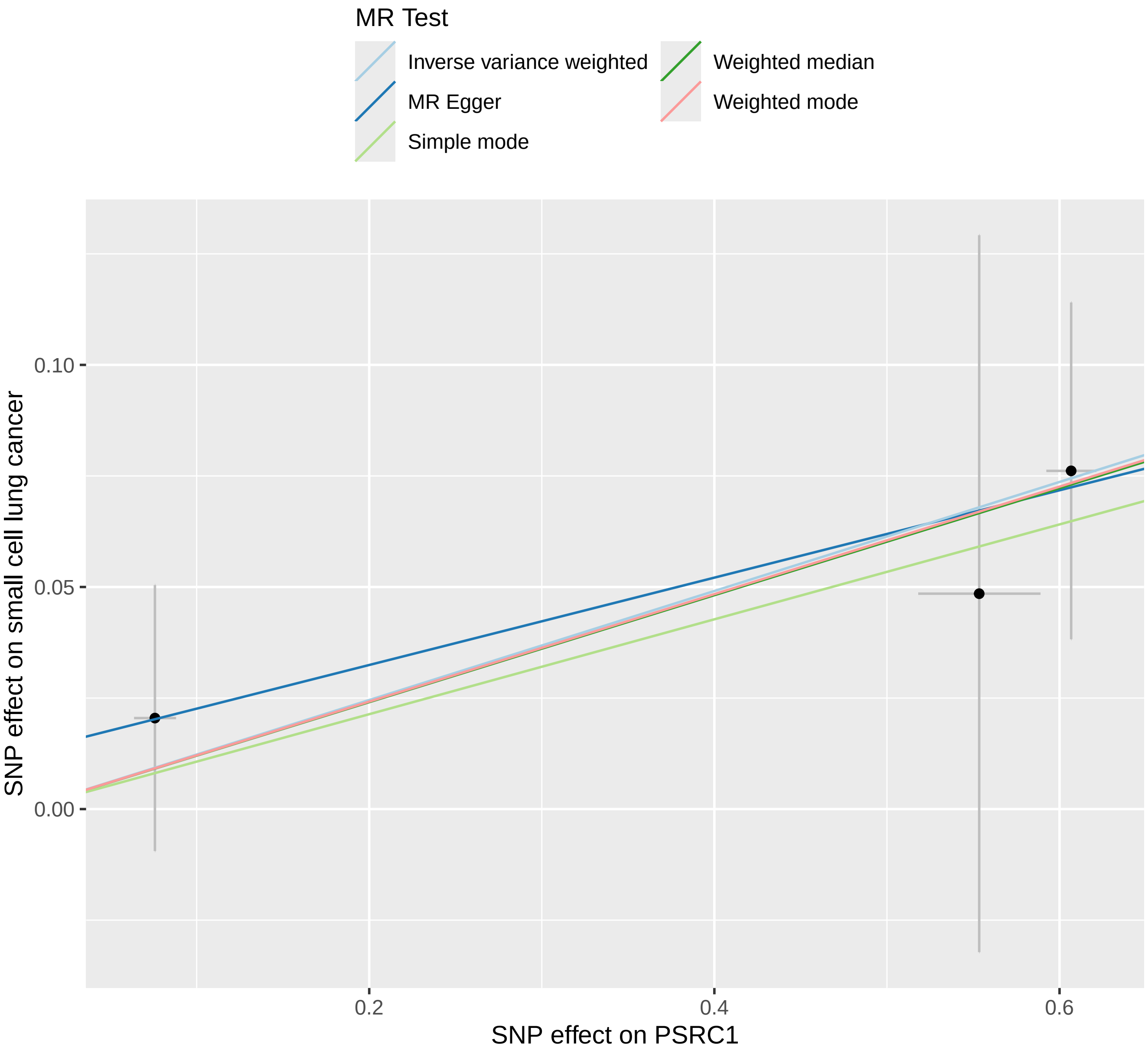
MR Test
Inverse variance weighted
MR Egger
Simple mode
Weighted median
Weighted mode
0.10
0.05
0.00
0.2
0.4
0.6
SNP effect on small cell lung cancer
SNP effect on PSRC1
