# Supplementary material for: Exploring potential therapeutic targets for small cell lung cancer based on transcriptomics combined with Mendelian randomization analysis
Source: Front Immunol. 2025 Jan 13;15:1464259. doi: 10.3389/fimmu.2024.1464259 (PMC11769988; doi:10.3389/fimmu.2024.1464259)
Supplement: Supplementary file 2 [file Image2.pdf]

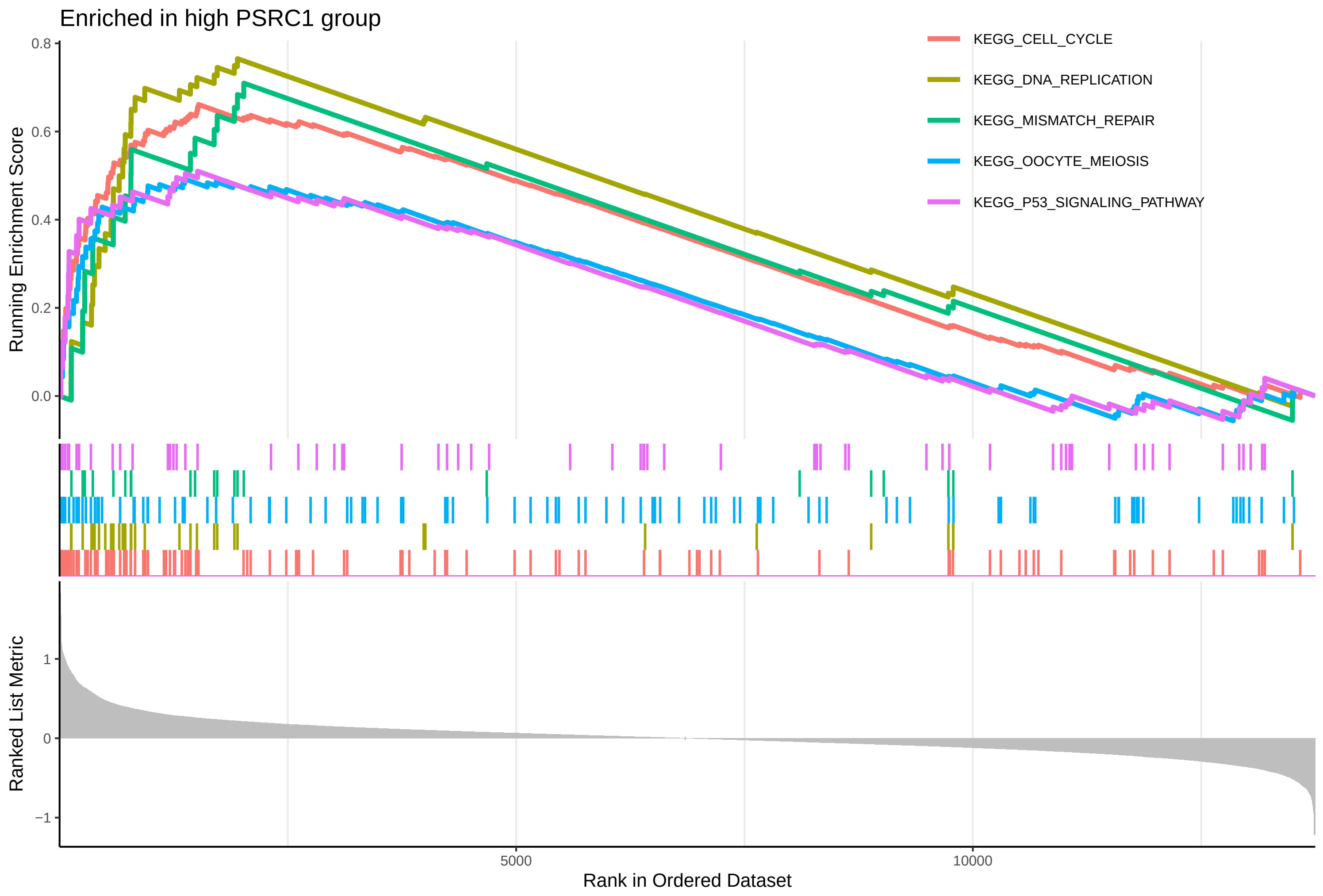
Enriched in high PSRC1 group
KEGG_CELL_CYCLE
KEGG_DNA_REPLICATION
KEGG_MISMATCH_REPAIR
KEGG_OOCYTE_MEIOSIS
KEGG_P53_SIGNALING_PATHWAY
Running Enrichment Score
0.8
0.6
0.4
0.2
0.0
Ranked List Metric
1
0
−1
5000
10000
Rank in Ordered Dataset

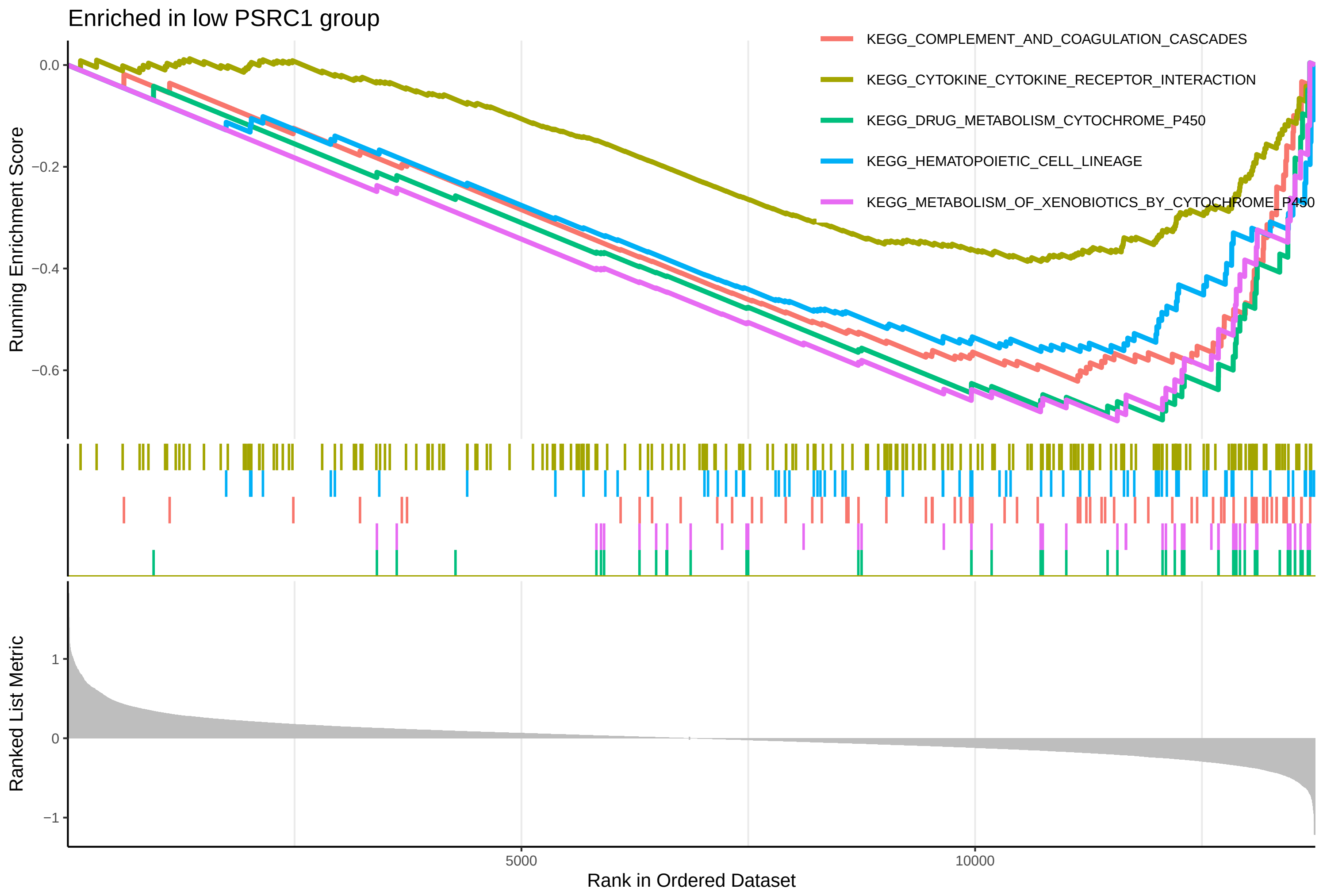
Enriched in low PSRC1 group
KEGG_COMPLEMENT_AND_COAGULATION_CASCADES
KEGG_CYTOKINE_CYTOKINE_RECEPTOR_INTERACTION
KEGG_DRUG_METABOLISM_CYTOCHROME_P450
KEGG_HEMATOPOIETIC_CELL_LINEAGE
KEGG_METABOLISM_OF_XENOBIOTICS_BY_CYTOCHROME_P450
Running Enrichment Score
0.0
−0.2
−0.4
−0.6
Ranked List Metric
1
0
−1
5000
10000
Rank in Ordered Dataset

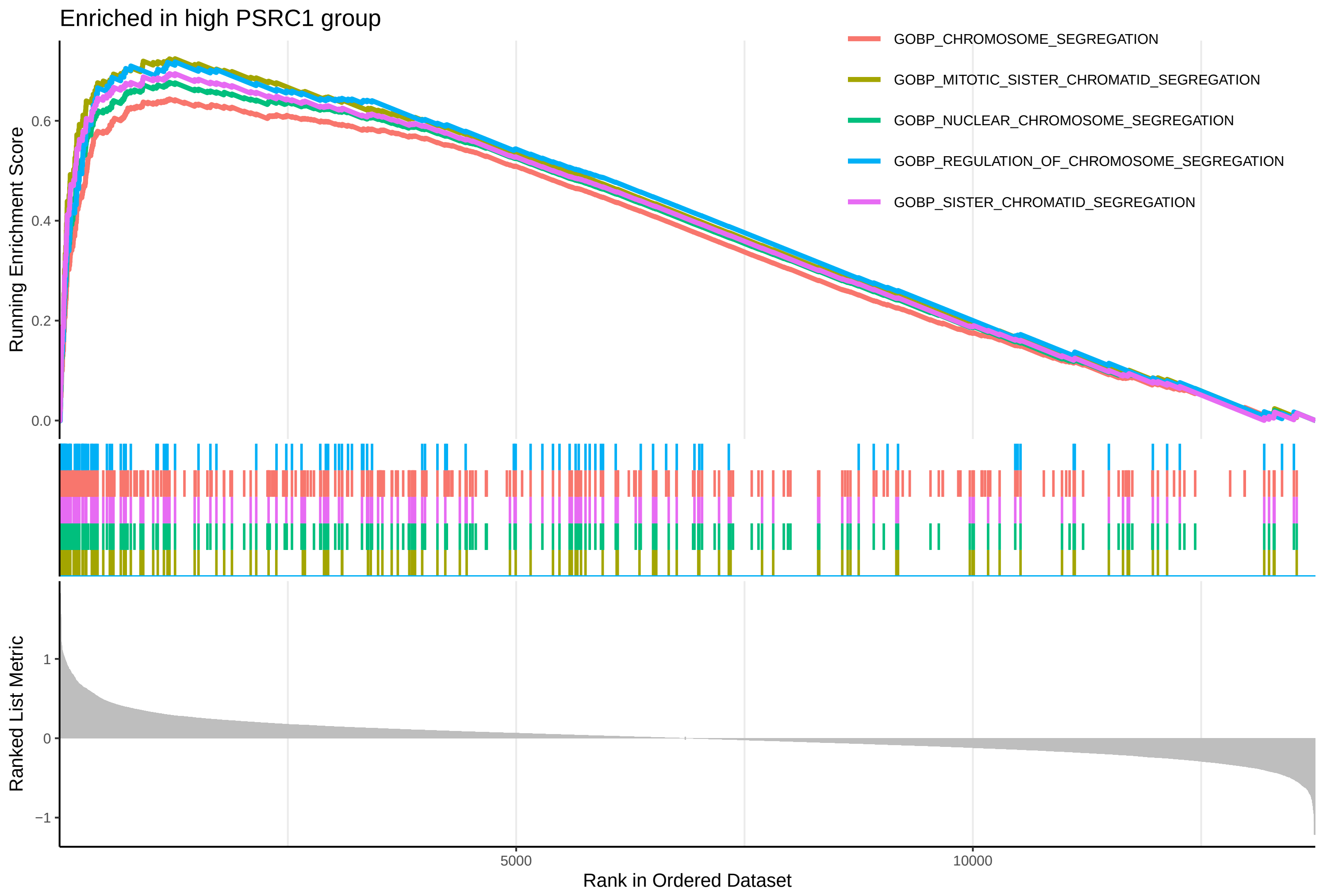
Enriched in high PSRC1 group
GOBP_CHROMOSOME_SEGREGATION
GOBP_MITOTIC_SISTER_CHROMATID_SEGREGATION
GOBP_NUCLEAR_CHROMOSOME_SEGREGATION
GOBP_REGULATION_OF_CHROMOSOME_SEGREGATION
GOBP_SISTER_CHROMATID_SEGREGATION
Running Enrichment Score
0.0
0.2
0.4
0.6
Ranked List Metric
1
0
−1
5000
10000
Rank in Ordered Dataset

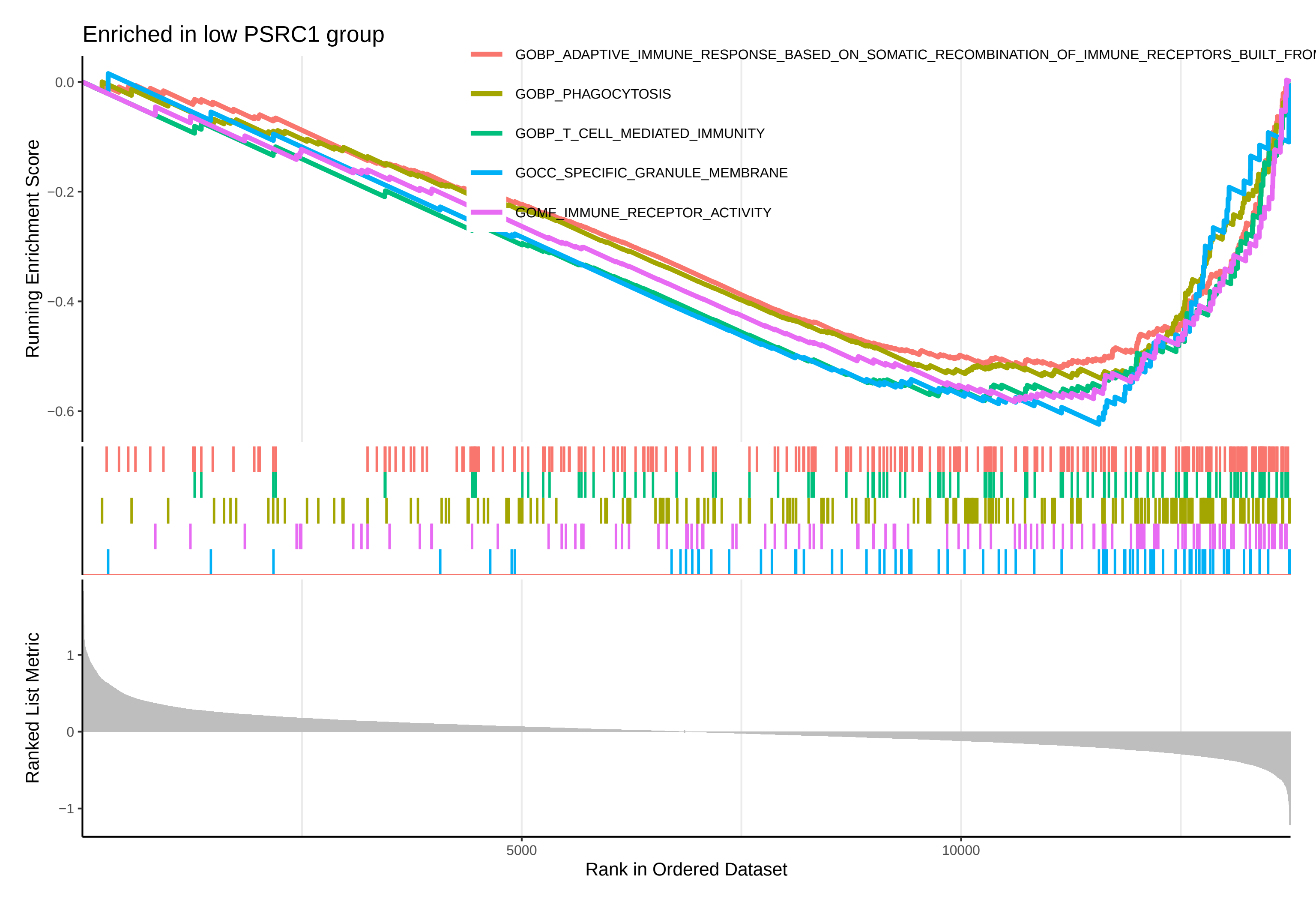

Enriched in low PSRC1 group
GOBP_ADAPTIVE_IMMUNE_RESPONSE_BASED_ON_SOMATIC_RECOMBINATION_OF_IMMUNE_RECEPTORS_BUILT_FROM
GOBP_PHAGOCYTOSIS
GOBP_T_CELL_MEDIATED_IMMUNITY
GOCC_SPECIFIC_GRANULE_MEMBRANE
GOMF_IMMUNE_RECEPTOR_ACTIVITY
Running Enrichment Score
0.0
−0.2
−0.4
−0.6
Ranked List Metric
1
0
−1
5000
10000
Rank in Ordered Dataset

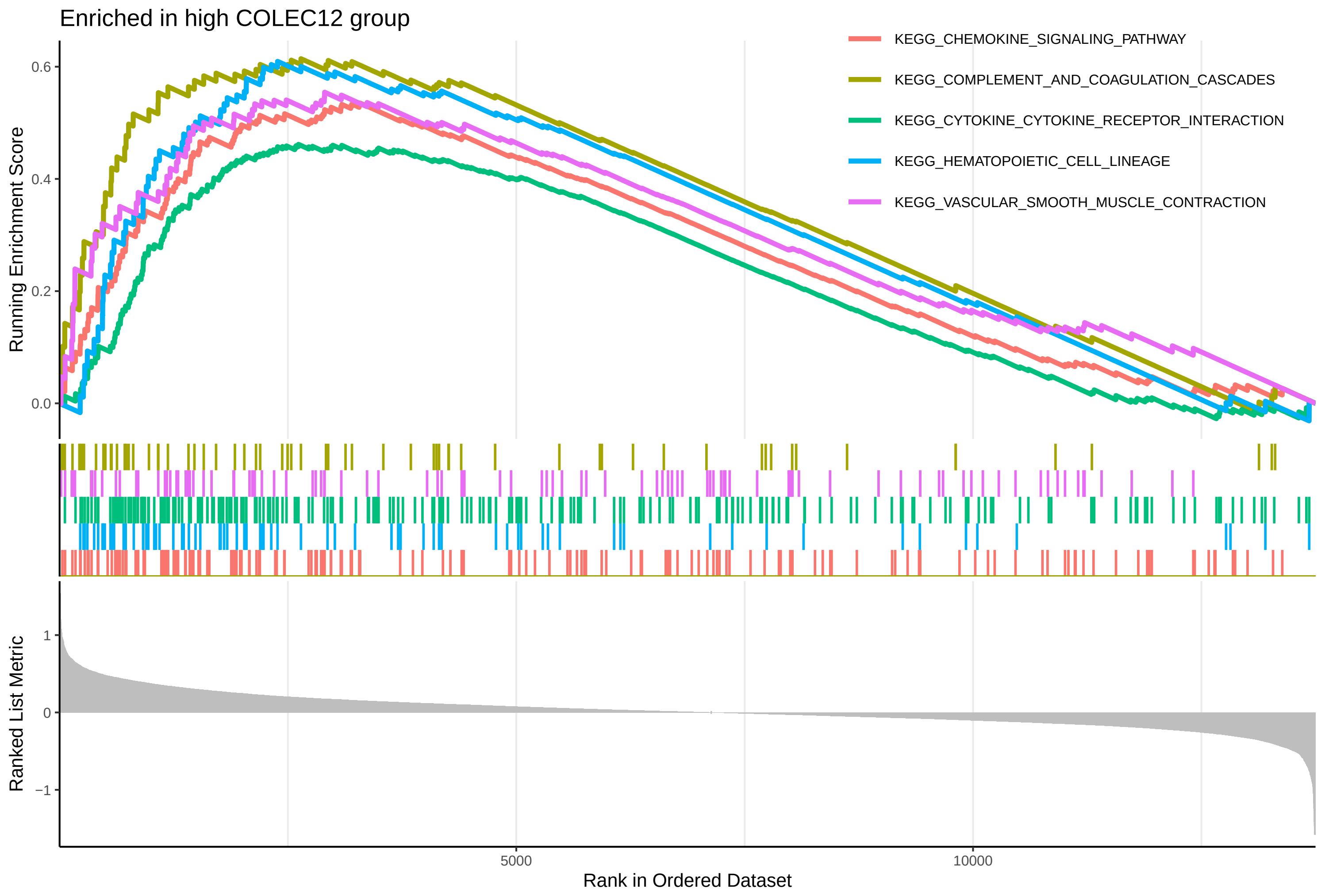

Enriched in high COLEC12 group
KEGG_CHEMOKINE_SIGNALING_PATHWAY
KEGG_COMPLEMENT_AND_COAGULATION_CASCADES
KEGG_CYTOKINE_CYTOKINE_RECEPTOR_INTERACTION
KEGG_HEMATOPOIETIC_CELL_LINEAGE
KEGG_VASCULAR_SMOOTH_MUSCLE_CONTRACTION
Running Enrichment Score
0.6
0.4
0.2
0.0
Ranked List Metric
1
0
−1
5000
10000
Rank in Ordered Dataset

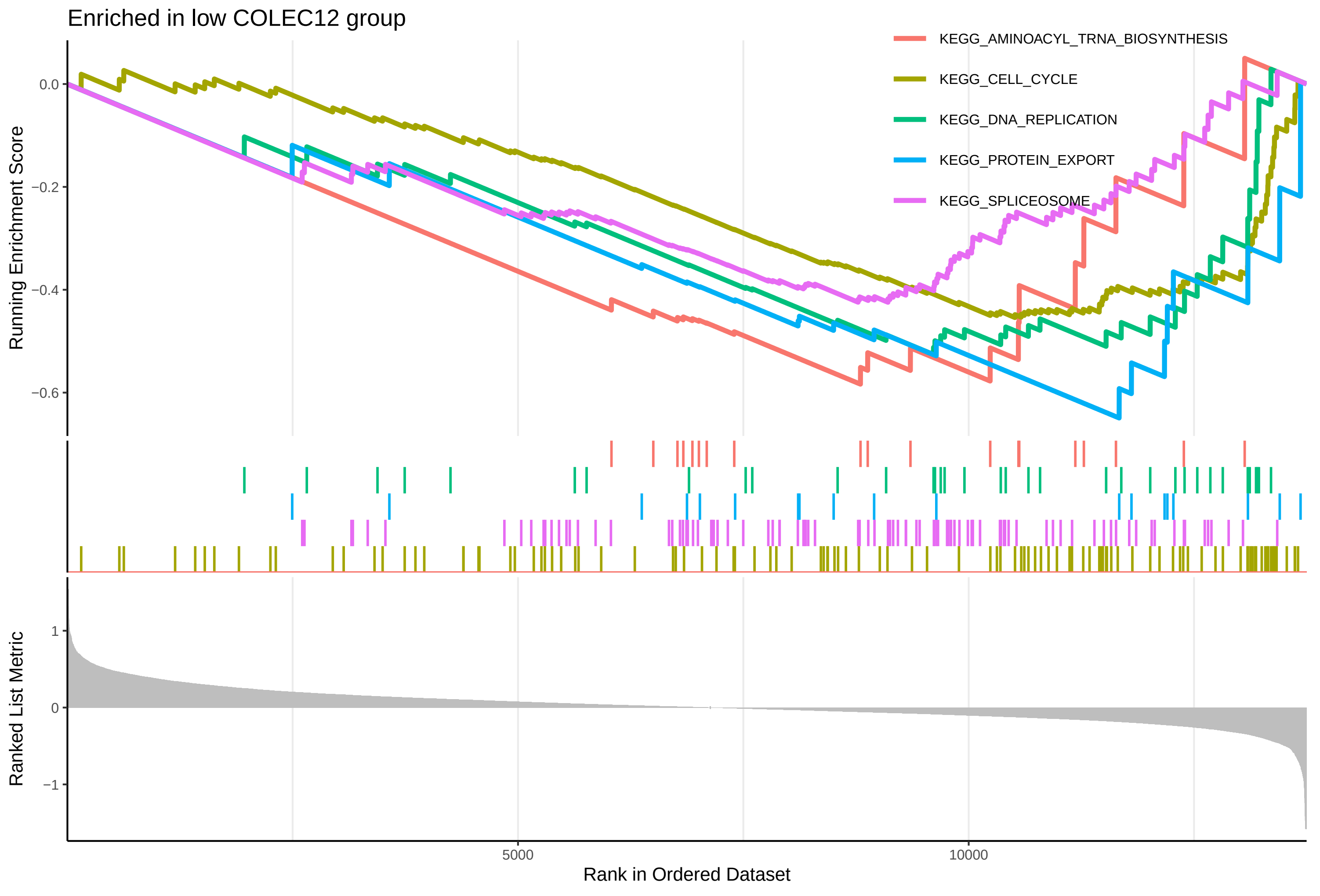
Enriched in low COLEC12 group
KEGG_AMINOACYL_TRNA_BIOSYNTHESIS
KEGG_CELL_CYCLE
KEGG_DNA_REPLICATION
KEGG_PROTEIN_EXPORT
KEGG_SPLICEOSOME
Running Enrichment Score
0.0
−0.2
−0.4
−0.6
Ranked List Metric
1
0
−1
5000
10000
Rank in Ordered Dataset

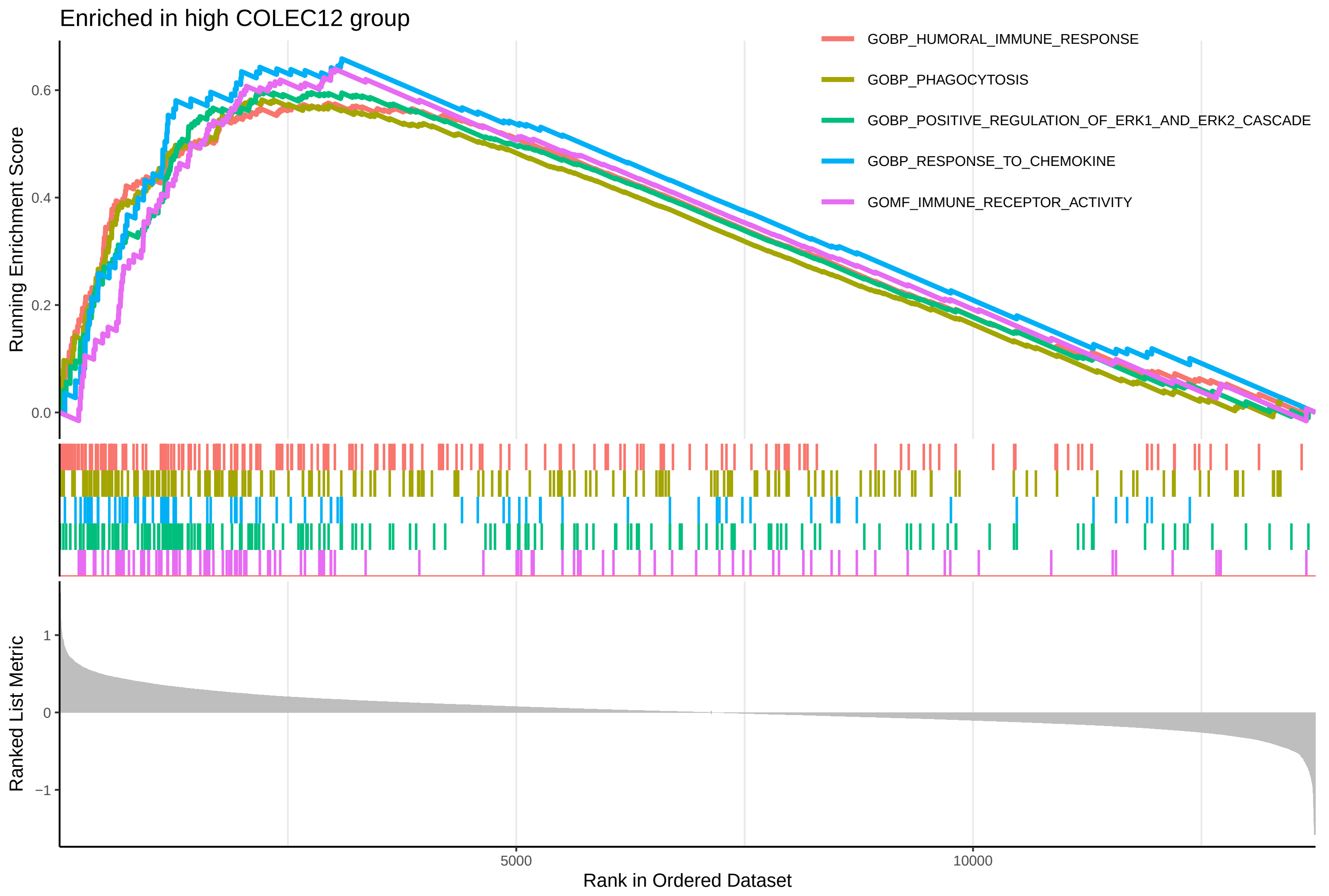

Enriched in high COLEC12 group
GOBP_HUMORAL_IMMUNE_RESPONSE
GOBP_PHAGOCYTOSIS
GOBP_POSITIVE_REGULATION_OF_ERK1_AND_ERK2_CASCADE
GOBP_RESPONSE_TO_CHEMOKINE
GOMF_IMMUNE_RECEPTOR_ACTIVITY
Running Enrichment Score
0.6
0.4
0.2
0.0
Ranked List Metric
1
0
−1
5000
10000
Rank in Ordered Dataset

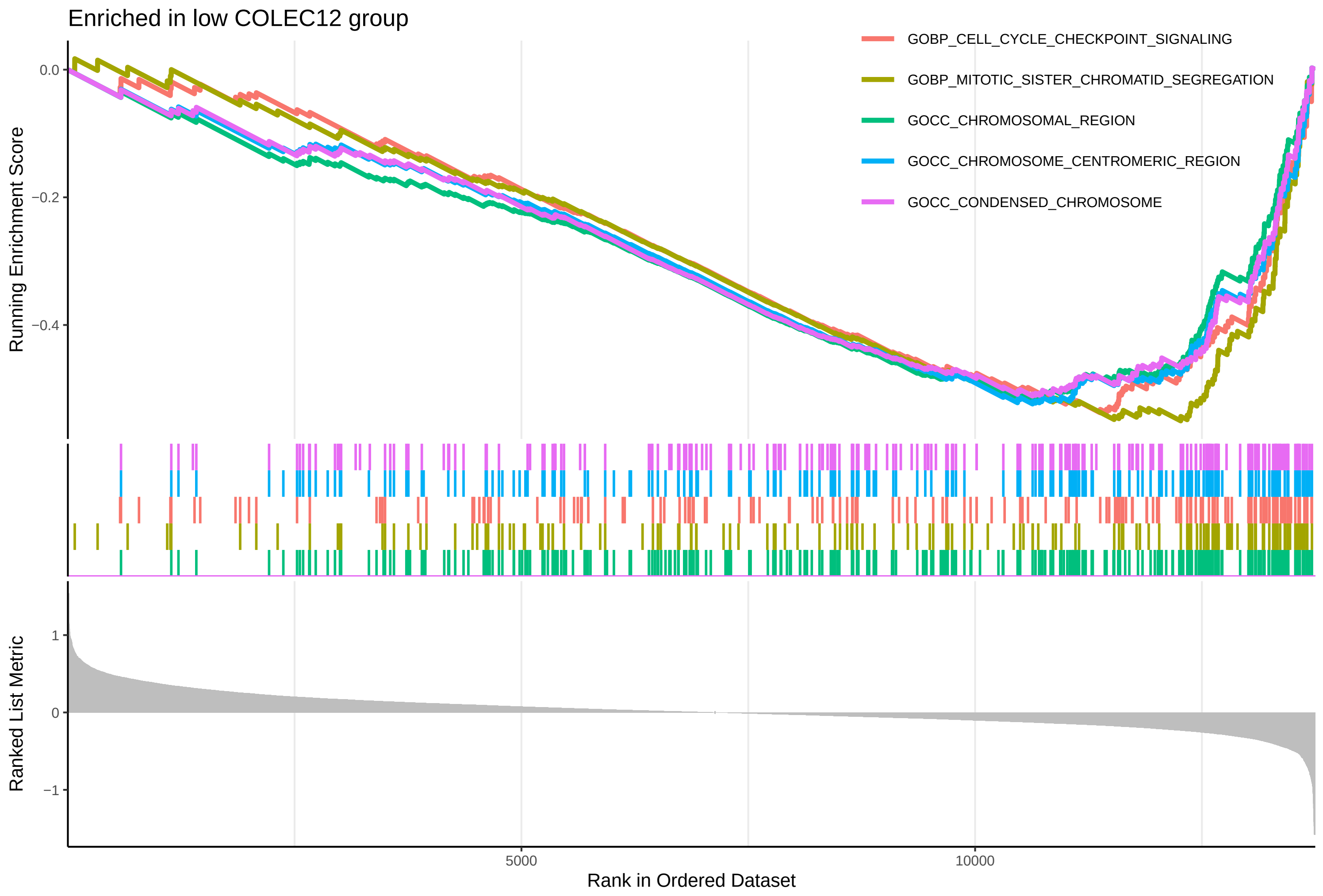
Enriched in low COLEC12 group
GOBP_CELL_CYCLE_CHECKPOINT_SIGNALING
GOBP_MITOTIC_SISTER_CHROMATID_SEGREGATION
GOCC_CHROMOSOMAL_REGION
GOCC_CHROMOSOME_CENTROMERIC_REGION
GOCC_CONDENSED_CHROMOSOME
Running Enrichment Score
0.0
−0.2
−0.4
Ranked List Metric
1
0
−1
5000
10000
Rank in Ordered Dataset
